# Glycoside hydrolase–mediated glucomannan catabolism in *Segatella copri*, a target of microbiota-directed foods for malnourished children

**DOI:** 10.1073/pnas.2521522122

**Published:** 2025-12-02

**Authors:** Cyrus Zhou, Matthew C. Hibberd, Evan M. Lee, Bo Pilgaard, Marlene Vuillemin, Emma Kiehn, Suzanne Henrissat, Marie A. Crane, Jiye Cheng, Lara Pfaff, Anne S. Meyer, Jesper Holck, Nicolas Terrapon, Juan J. Castillo, Garret Couture, Carlito B. Lebrilla, Dmitry A. Rodionov, Michael J. Barratt, Bernard Henrissat, Jeffrey I. Gordon

**Affiliations:** ^a^The Edison Family Center for Genome Sciences and Systems Biology, Washington University School of Medicine, St. Louis, MO 63110; ^b^The Newman Center for Gut Microbiome and Nutrition Research, Washington University School of Medicine, St. Louis, MO 63110; ^c^Department of Pathology and Immunology, Washington University School of Medicine, St. Louis, MO 63110; ^d^Department of Biotechnology and Biomedicine, Technical University of Denmark, Kgs. Lyngby DK-2800, Denmark; ^e^Architecture et Fonction des Macromolécules Biologiques, CNRS, Aix-Marseille Université, Marseille F-13288, France; ^f^Department of Chemistry, University of California, Davis, CA 95616; ^g^Infectious and Inflammatory Disease Center, Sanford Burnham Prebys Medical Discovery Institute, La Jolla, CA 92037

**Keywords:** gut microbiome-directed therapeutics, prebiotic discovery, carbohydrate-active enzymes, polysaccharide utilization loci, *Segatella copri*

## Abstract

Evidence is emerging that perturbed postnatal gut microbiota development is causally related to childhood undernutrition. Clinical trials in undernourished Bangladeshi children found that a polysaccharide-rich, microbiota-directed complementary food (MDCF-2) designed to repair this perturbation produced superior ponderal and linear growth compared to a standard ready-to-use supplementary food. Subsequent analyses disclosed several candidate bioactive polysaccharides in the MDCF and their bacterial targets, notably strains of *Segatella copri* that possess carbohydrate-active enzymes (CAZymes) organized into polysaccharide utilization loci (PULs) targeting these glycans. A Bangladeshi *S. copri* isolate (BgF5_2) containing these PULs metabolized MDCF-2 glycans and promoted MDCF-dependent weight gain in a gnotobiotic mouse model emulating the clinical trials. Identifying prebiotic mixtures that mimic the effects of MDCF-2 would offer new options for treatment and prevention. Here, we describe a CAZyme-based approach to characterize the effects of glucomannan, a component of MDCF obtainable from sustainable sources, on growth and gene expression in *S. copri* BgF5_2 in vitro and in gnotobiotic mice. Biochemical characterization of purified CAZymes expressed by two of its MDCF-2 and glucomannan-targeted PULs disclosed a multifunctional GH26|GH5_4 CAZyme, inducible by glucomannan, that degrades several bioactive MDCF-2 glycans; glucomannan, arabinoxylan, xyloglucan, and mixed-linkage β-glucan. Our data suggest that this CAZyme functions as a multisubstrate “sentinel” that can produce diverse oligosaccharides from a variety of β-linked glycans, with each oligosaccharide able to induce corresponding PULs and non-PUL enzymes. This observation, plus the restricted distribution of the multifunctional CAZyme among *S. copri* strains, may partially explain strain responsiveness to MDCF-2.

## Significance

Preclinical and clinical studies have identified candidate bioactive polysaccharides in a microbiota-directed food used to treat acutely malnourished Bangladeshi children during complementary feeding. Findings related to this microbiota-directed complementary food (MDCF-2) may enable the design of prebiotic formulations that mimic its effects and offer more flexibility for administration and precise dosing. Using glucomannan as an example, we present an approach for manipulating *Segatella copri*, a prominent bacterial MDCF-2 “target,” using glycans from sustainable sources that combines comparative genomic analyses of its carbohydrate-active enzymes, their biochemical characterization, and their expression in vitro and in gnotobiotic mice containing human gut microbes. The results reveal a unique, multifunctional enzyme whose expression is induced by glucomannan and that catabolizes it plus other MDCF-2 polysaccharides.

Sixty million infants and children worldwide currently suffer from impaired ponderal growth [wasting, defined anthropometrically by weight-for-length z-score (WLZ) ≤−2)] (1–3); 45 million have moderate acute malnutrition (MAM), for which there is no generally recommended protocol for treatment, while 15 million have severe acute malnutrition (SAM) where administration of ready-to-use-therapeutic-foods is typically stopped when anthropometric recovery is achieved (WLZ ≥−2) (3). At least 150 million children have impaired linear growth (stunting). Beyond accounting for half of all mortality in children under the age of five, childhood undernutrition leads to long-term growth impairments, immune and metabolic dysfunction, and altered central nervous system development (4). Current treatments have improved mortality but have had limited effectiveness in meeting global health goals for curtailing undernutrition (4).

Our prior studies have demonstrated a causal link between impaired development of the gut microbiota and acute malnutrition (5, 6). When introduced into just-weaned germ-free mice, gut microbial communities from undernourished children led to impaired lean body mass gain, altered bone growth, as well as immune and metabolic abnormalities when compared to mice colonized with communities from age-matched healthy children (e.g., ref. 7). Application of machine learning methods to the transplanted communities revealed “growth-discriminatory” bacterial taxa (organisms whose abundances correlated with the growth phenotypes of recipient gnotobiotic animals). A number of these growth-discriminatory taxa are not appropriately represented in the fecal microbiota of children with MAM and SAM during the weaning period. Screening “complementary” foods, given during the weaning period to Bangladeshi children, for their capacity to influence the fitness (abundance) or expressed functions of growth-associated taxa in mice colonized with microbiota from undernourished Bangladeshi children led to the identification of several candidate food ingredients that were combined into a “microbiota-directed complementary food” formulation (MDCF-2) (8). The efficacy of MDCF-2 was tested in randomized controlled clinical trials where 12 to 18-mo-old Bangladeshi children suffering from either primary MAM or having just recovered from SAM to a state of “post-SAM” MAM, received dietary supplementation with either MDCF-2 or an existing ready-to-use supplementary food (RUSF) twice-daily for 3 mo (9, 10). Consumption of MDCF-2 produced superior growth rates and greater changes in plasma protein biomarkers and mediators of musculoskeletal and CNS development, immune function, plus other facets of physiologic and metabolic regulation despite the fact that MDCF-2 had 15% lower caloric density than RUSF (11).

Further assessments of the bioactive components of MDCF-2 and their microbial targets were based on analyses of the encoded and expressed functions of bacterial metagenome-assembled genomes (MAGs) that had been identified by shotgun sequencing of DNA isolated from serially collected fecal samples from study participants (9, 11). Elevated expression of genes in carbohydrate metabolism pathways correlated with i) the representation of glycans enriched in, or unique to, MDCF-2 compared to RUSF and ii) the magnitude of the weight gain in MDCF-2 treated participants (11). The majority of these transcriptional responses were attributed to two MAGs (Bg0018 and Bg0019) assigned to *Segatella copri* (previously named *Prevotella copri*; ref. 12). The abundances of these *S. copri* MAGs were positively correlated with anthropometric measures of ponderal growth and they encode polysaccharide utilization pathways predicted to be relevant to degradation of MDCF-2 glycans.

Polysaccharide utilization loci (PULs) are clusters of genes distributed throughout the genomes of members of Bacteroidota, including *S. copri*; their encoded proteins mediate detection, import, and breakdown of a specific glycan or set of glycans (13–15). *S. copri* MAGs Bg0018 and Bg0019 share seven PULs that are highly conserved (i.e., ≥90% amino acid identity per gene and identical genomic organization) and three PULs designated as present but “structurally distinct” (i.e., displaying genomic but not necessarily functional differences). An extensive culturing effort based on fecal samples from Bangladeshi trial participants yielded *S. copri* isolates with genomic similarity to these two MAGs; the greatest similarity was observed in *S. copri* strain BgF5_2 (98% overall nucleotide sequence identity with *S. copri* MAG Bg0019; 11). Advancement of these isolates to a gnotobiotic mouse model of mother-to-offspring microbiota transmission that involved cultured, age- and growth-associated Bangladeshi bacterial strains provided direct evidence that *S. copri* plays a key role in metabolizing MDCF-2 glycans, promoting weight gain in an MDCF-2-dependent fashion, and regulating activities of metabolic and cell–cell signaling pathways in intestinal epithelial cell lineages (16).

Analysis of the polysaccharide composition of MDCF-2 and RUSF revealed that MDCF-2 contained significantly more β-mannan and pectic galactan—glycans that are predicted to be substrates of the majority (5/7) of the PULs conserved between MAGs Bg0018 and Bg0019. Furthermore, differences in the abundances of specific glycosidic linkages in the feces of trial participants before and after MDCF-2 treatment could be explained in part by the predicted specificities of expressed CAZymes encoded by these conserved PULs (11).

Together, these findings support the hypothesis that combinations of glycans representing the bioactive components of MDCF-2 could be used as prebiotics to treat undernourished children based on their ability to support the fitness or expressed functions of key growth-promoting members of the developing microbiota. The advantage of this approach would be several-fold: i) In principle, prebiotics could be added (e.g., as prepackaged powders) to various culturally acceptable, locally available foods and used in lieu of the need to manufacture and distribute shelf-stable, organoleptically acceptable therapeutic food formulations, ii) improved efficacy based on better knowledge of and ability to control dosage, and iii) the ability to develop synbiotics (combinations of prebiotic glycans and their target bacterial strains) to treat children who initially fail to respond to treatment with microbiota-directed therapeutic foods and/or lack critical growth-associated and prebiotic-responsive strains.

In theory, the ability of a given bacterium to break down a given glycan corresponds to the substrate specificities of the CAZymes encoded by its genome. Whether theoretical or estimated, the diversity of glycan structures is much greater than the diversity of protein folds in CAZymes (17, 18). The CAZy database groups families and subfamilies of CAZymes based on their amino acid sequence similarity and the 3-D structure of independently folding protein modules (19). Combined with knowledge of biochemically validated substrate specificities, the CAZyme-based approach becomes a powerful tool for propagating carbohydrate-related functional predictions onto enzymes that have not been previously characterized.

With these considerations in mind, we reasoned that one approach to identifying prebiotic candidates resembling MDCF-2-associated glycan structures would be to first consider the purity of available compounds with these structures (e.g., from the byproduct streams of food manufacture), the scale at which they are being or could be produced, and data about human consumption (e.g., as components of commercial foods). Candidates would then be subjected to a multistep analytic effort that included initial assessment of their effects on the growth and patterns of CAZyme gene expression in cultured strains representing the therapeutic target (e.g., *S. copri* BgF5_2). Biochemical analyses would follow using purified proteins encoded by CAZyme genes whose expression was shown to be regulated in vitro in response to the prebiotic candidate. The capacity of these purified enzymes to degrade structures in the candidate prebiotic as well as in other related available glycans (e.g., those with variations in branched chains) would then be determined. Finally, prebiotic candidates emerging from these in vitro analyses would be tested in gnotobiotic mice colonized postnatally with consortia of age- and growth-discriminatory gut bacterial strains, including the target organism(s), to determine the effect of the glycan alone, or embedded in a food matrix, on the target(s) as well as other community members and the host. In this report, we illustrate this approach for evaluating candidate prebiotics using a purified glycan present in MDCF-2 that is commonly consumed and available at scale at high purity.

## Results

### Identification of Predicted Glucomannan-Degrading CAZymes in Ponderal Growth-Associated *S. copri* MAGs.

The four complementary foods present in MDCF-2 are green banana (plantain), chickpea flour, peanut flour, and soybean flour. Glycan composition analyses of these four ingredients by ultrahigh performance chromatography-triple quadrupole mass spectrometry (UHPLC-QqQ-MS) (11, 20–23) revealed that soybean flour, a rich source of candidate bioactive glycans, contains glucomannan. Glucomannan is a water-soluble polysaccharide consisting of repeating glucose and mannose units linked via β-1,4-glucosidic and β-1,4-mannosidic bonds. The stoichiometry of the monomers can vary according to its botanical source. Humans lack the CAZymes necessary to cleave either of these two β-1,4 linkages (24); therefore, host utilization of this glycan must be initiated by gut microbial enzymes. Glucomannan is commonly consumed by humans (e.g., as a gelling agent in commercial food products) and is available in purified form from sustainable sources in bulk quantities at relatively low cost, making it an attractive prebiotic candidate.

**Fig. 1.**
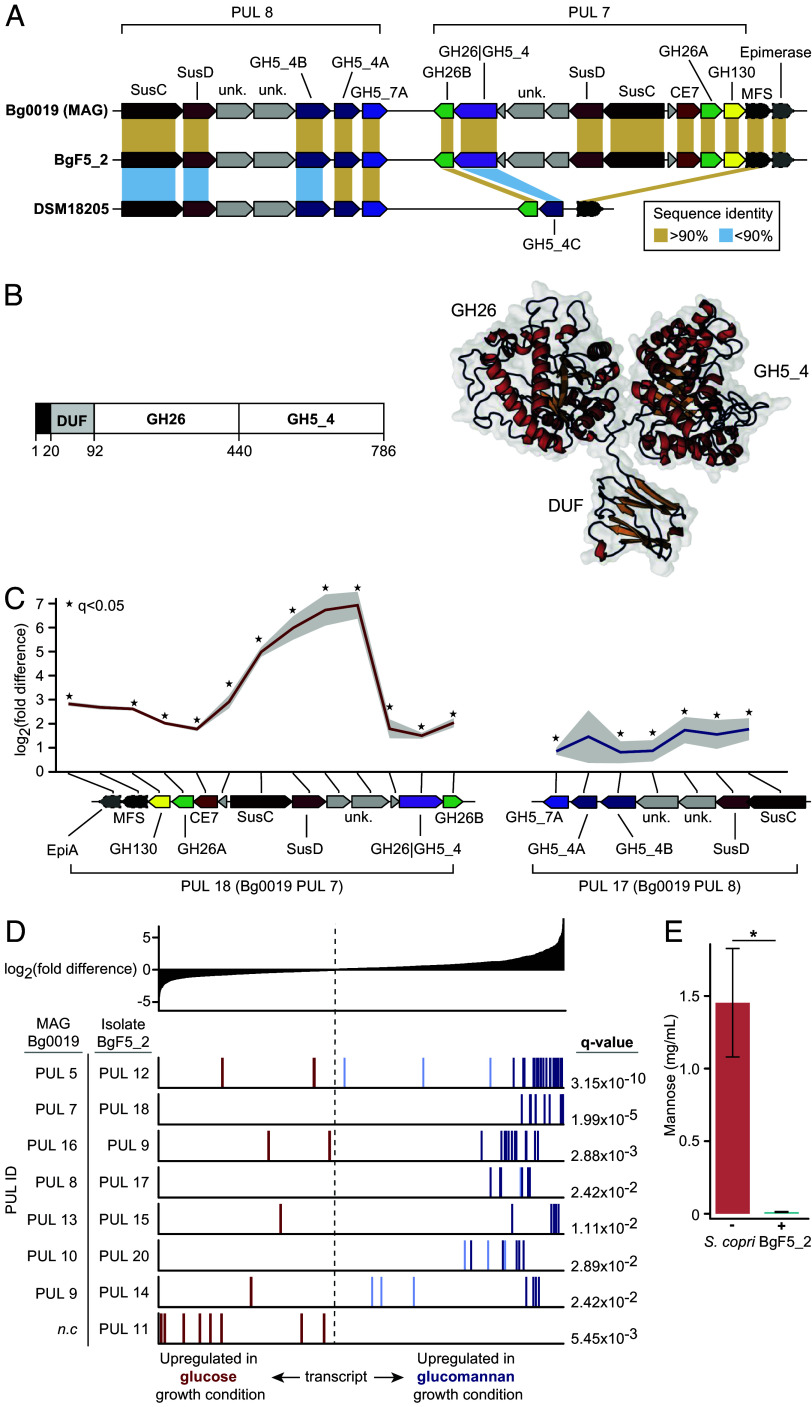
Structures and transcriptional responses of *S. copri* polysaccharide utilization loci to glucomannan in vitro. (*A*) Schematic of the genes encoded in homologs of two PULs represented in the *S. copri* MAG Bg0019 that is positively associated with WLZ, a genetically and functionally similar cultured Bangladeshi *S. copri* isolate, BgF5_2, and the *S. copri* type strain, DSM18205. (*B*) GH26|GH5_4 enzyme. Left portion of the panel illustrates the approximate boundaries of its domains (residues 1 to 19 denote the signal peptide, while DUF represents a domain of unknown function). The right portion of the panel presents an AlphaFold predicted structure without the signal peptide. By analogy to so-called BACON domains (25), we presume the role of the DUF is that of a spacer allowing the catalytic modules to act at a distance from the outer membrane. (*C*) Differential expression (log_2_ fold difference ± SE, gray ribbons) of genes constituting the homologs of the glucomannan degradation PUL 7 (*Left*) and the adjacent PUL 8 (*Right*) in isolate BgF5_2 during growth in a carbohydrate-deficient medium (PCDM) supplemented with versus without glucomannan. *q < 0.05 (DESeq2) (*D*) Differential expression of PULs considered as multigene transcriptional units. PUL identifiers for the WLZ-associated *S. copri* MAG Bg0019 and the homologous PUL in the BgF5_2 isolate are indicated on the left. The dashed line indicates the center of the distribution of log_2_ fold-differences from elevated in the glucose-supplemented condition (*Left*) to elevated in the glucomannan-supplemented condition (*Right*). Each set of vertical bars correspondi to the gene in a significantly upregulated PUL (q < 0.05, GSEA). The location along the horizontal line indicates their position in the differential expression distribution shown at the top of the panel. The color of the vertical bars (genes) are based on their differential expression (DESeq, dark colors indicate q < 0.05), light colors indicate q > 0.05) *n.c.,* not conserved in MAG Bg0019. (*E*) UHPLC–QqQ-MS quantification of total mannose present in glucomannan-supplemented PCDM collected from mid-log phase cultures of BgF5_2 or the equivalent timepoint in medium-only controls. Error bars represent SD. *P < 0.05, Student’s *t* test.

CAZymes known to cleave β-1,4-mannosidic and β-1,4-glucosidic linkages present in glucomannan belong to glycoside hydrolase families GH26 and GH5. Therefore, we searched the *S. copri* proteome for GH26 and GH5 homologs using a 40% identity cut-off and determined their PUL membership, the latter using an algorithm underlying a PUL database (PULDB; 15). Homologs of these two GH families were confined to two PULs that are conserved in MAGs Bg0018 and Bg0019. Using a PUL numbering convention based on MAG Bg0019, these loci have been designated PUL 7 and PUL 8 (Fig. 1*A* and Table 1).

**Table 1.** PULs of *S. copri* MAG Bg0019 and isolate BgF5_2

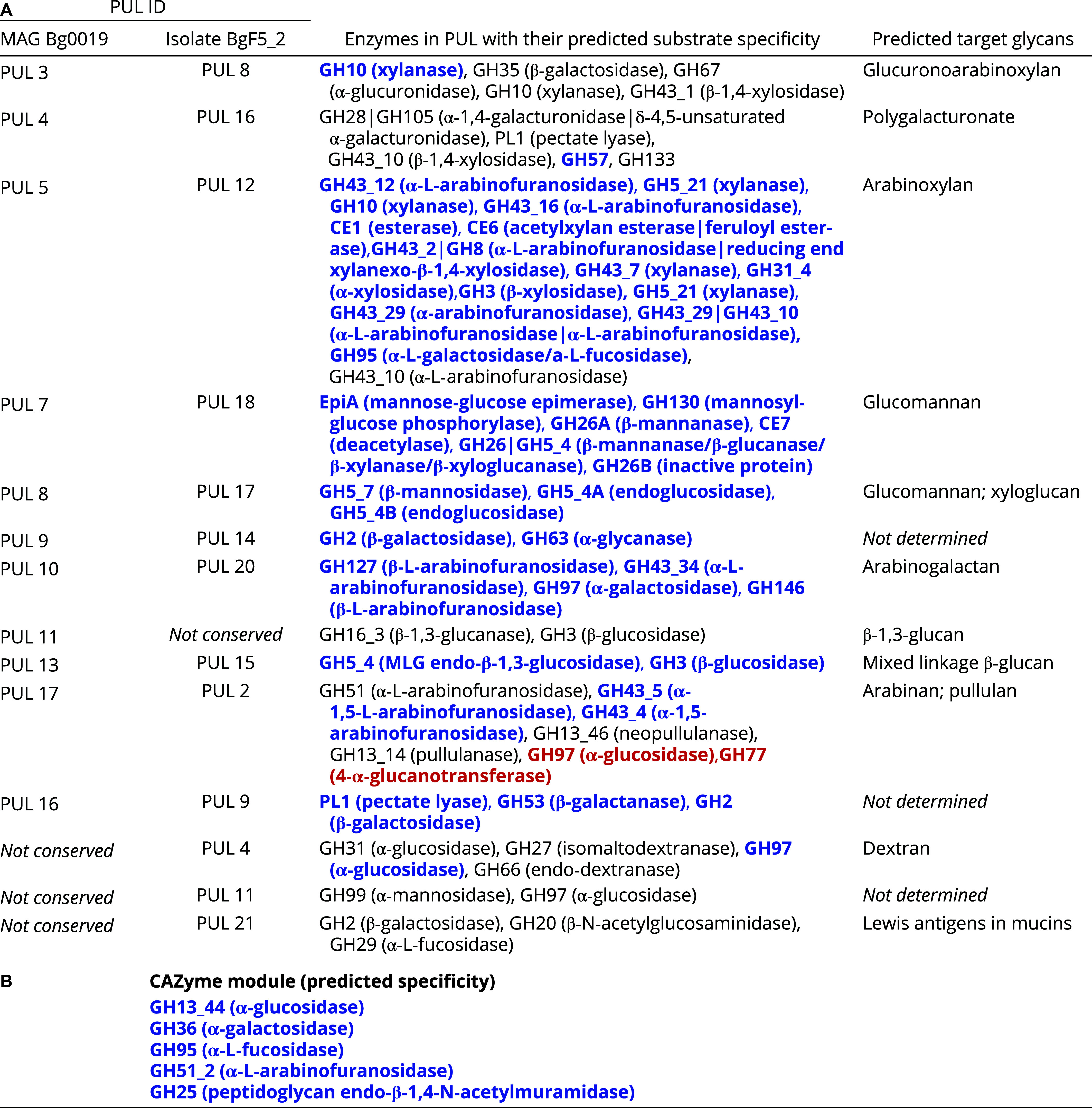

(A) PUL correspondence between BgF5_2 and Bg0019, including component CAZymes, predicted enzymatic activities and substrates. (B) Glucomannan-responsive CAZymes located outside predicted PUL boundaries. Individual CAZyme are colored by their differential regulation in vitro in response to glucomannan supplementation: blue (upregulation), red (downregulation), or black (not significantly differentially regulated).

PUL 7 is predicted to extend across 11 open reading frames in MAG Bg0019 and encodes a SusC/D transporter and five CAZymes: an unusual bimodular protein containing a N-terminal domain from family GH26 and a C-terminal domain from family GH5_4 (referred to here as GH26|GH5_4) (Fig. 1*B*); two proteins from family GH26 (GH26A and GH26B); one member of family GH130; and one predicted CE7 esterase (Fig. 1*A* and Dataset S1). Intriguingly, the GH26B protein has mutations at each of the two catalytic residues identified in family GH26 (26) and is thus predicted to be inactive as a glycoside hydrolase. These two inactivating mutations are conserved in the GH26 homologs of both MAG Bg0019 and Bg0018, plus 62 additional *S. copri* isolate genomes and MAGs retrieved from PULDB (Dataset S2), suggesting that the GH26B homologs have undergone an evolutionarily conserved shift to a nonglycoside hydrolase function.

Signal peptide-based subcellular localization predictions (*Methods*) of the CAZymes specified by PUL 7 suggest that GH26|GH5_4 and GH26B possess SPaseI cleavage sites. Neither protein possesses a Lipoprotein Export Signal (LES) motif originally defined in *Capnocytophaga canimorsus* (27). No cysteine lipid anchor appears immediately after the predicted SPaseI cleavage site in either enzyme. However, a region of 60 amino acids (for GH26|GH5_4) or 30 amino acids (for GH26B) separate their SPaseI cleavage site and catalytic domains. Both regions contain a conserved Q-(T/V)-D-G-(D/V) sequence similar to the Q-A-(D/E)_2_ motif reported to direct lipoprotein export in *C. canimorsus*. Given these considerations, we predict that both GH26|GH5_4 and GH26B are bound to the outer membrane through lipid anchorage or a similar mechanism and face the extracellular milieu to perform their respective functions. The other PUL 7 CAZymes are predicted to be periplasmic except for the GH130 protein which is predicted to be cytosolic.

PUL 8 is adjacent to PUL 7 and extends across seven open reading frames in MAG Bg0019 (Fig. 1*A*). It encodes a SusC/D transporter, two CAZymes from family GH5_4 (GH5_4A and GH5_4B) and one from family GH5_7. In PUL 8, GH5_4A is predicted to be located on the outer membrane. GH5_4B and GH5_7 are predicted to be periplasmic.

Of the putative PUL 7 and PUL 8 CAZymes exposed to the external milieu where initiation of glycan degradation occurs, the 786 amino acid bimodular GH26|GH5_4 protein was of special interest as its N- and C-terminal domains (excluding an N-terminal Domain of Unknown Function; Fig. 1*B*) are in theory capable of cleaving the β-mannosidic and β-glucosidic bonds present in glucomannan, respectively. The GH26|GH5_4 protein has not been reported previously to be associated with human-derived microbes but has been observed in the thermophile *Acetivibrio thermocellum* (28) and in several rumen-resident organisms (29–32). However, CAZymes of this type have been recently shown to initiate the degradation of glycans not typically viewed as bacterial growth substrates (33). These earlier biochemical characterizations suggest that the human microbiota-associated homolog may also cleave a wide range of β-linked glycans.

We performed a detailed comparative genomics analysis of the MAG Bg0019 GH26|GH5_4 enzyme, focusing on its phylogenetic and geographic distribution and sequence conservation. Initial homology-based searches spanning 1,889 genome sequences (including 101 MAGs) and 222 Bacteroidota genera disclosed that this enzyme is only found in its complete bimodular form in organisms assigned to the genus *Segatella/Prevotella* (Dataset S2). Therefore, we focused our analysis on a set of genome sequences including 484 MAGs, 369 reference genomes and 58 isolate genomes all assigned to *Segatella/Prevotella*. A marker gene-based phylogenetic tree was constructed from these genomes (*SI Appendix*) and the results of homology searches of the MAG Bg0019 GH26|GH5_4 protein sequence against each genome were aligned to the tree. The results disclosed that the GH26|GH5_4 enzyme is largely restricted to genomes assigned to *S. copri*, although it is also encoded by representatives of other phylogenetically distinct species including *Prevotella bryantii*, *Prevotella baroniae*, *Prevotella herbatica,* and *Prevotella paludivivens* (*SI Appendix*, Fig. S1). Conservation of the full-length enzyme within *S. copri* ranged from 83-98% (amino acid identity) and dropped to 56 to 61% within non-*S. copri* species (Dataset S2). Notably, the presence of this enzyme within *S. copri* was not universal; it was found in most MAG and isolate genomes assigned to Clades A and D, while being nearly absent from genomes assigned to Clades C and B (34) and from the *S. copri* type strain DSM 18205. Within Clade A, isolates related to the ponderal growth (WLZ)-associated MAGs Bg0019/Bg0018 and BgpSM0038/BgpSM0080 identified in our previous clinical trials of MDCF-2 (9, 11) contained the enzyme. In light of these findings, we examined the geographical distribution of genomes with this enzyme. To control for the differential yield of isolates classified as *S. copri* versus other taxa in each country, we restricted the analyzed genomes to those falling within the *S. copri/P. hominis* clade (*SI Appendix*, Fig. S1). Cultured Bangladeshi *S. copri* isolates exhibited a GH26|GH5_4 prevalence of 46.3% (25/54 isolates) whereas *S. copri* isolates from China and the United States displayed a greater prevalence [20/27 isolates (74.1%) and 94/108 (87.0%), respectively]. Notably, high quality MAGs assembled from a number of Bangladeshi observational and MDCF interventional studies displayed a lower prevalence [26/111 isolates (23.4%)]. These results indicate that presence and conservation of this enzyme varies across different human gut bacterial species, across different strains comprising different *S. copri* clades, and across different countries.

### Effects of Glucomannan on the *S. copri* Transcriptome.

Table 1 lists our initial predictions of the glycans that can be targeted by the PULs in MAG Bg0019 and the corresponding PULs in the cultured *S. copri* BgF5_2 isolate based on their CAZyme content. To characterize the transcriptional responses to glucomannan in *S. copri* strain BgF5_2, we cultured this organism in a carbohydrate-deficient defined medium (PCDM; 11, 35) supplemented with either glucomannan or glucose as the sole carbon source. In parallel, we assayed the growth phenotype of the *S. copri* type strain DSM 18205; this strain lacks a conserved PUL 7 analog and the bimodular enzyme but possesses six other PULs homologous to those conserved between MAGs Bg0019 and Bg0018, albeit less so than in BgF5_2 (ref. 11 and Dataset S1*B*). While both *S. copri* BgF5_2 and the DSM 18205 type strain were able to grow in PCDM-glucose, only *S. copri* BgF5_2 was able to grow in PCDM-glucomannan (Dataset S1*C*).

The transcriptome of *S. copri* BgF5_2 was characterized at mid-log phase when grown in PCDM-glucose or PCDM-glucomannan (1.5 % w/v). A total of 547 genes exhibited significant differential expression (*Methods*) in the different growth media of which 333 were upregulated in PCDM-glucomannan (Dataset S3*A*). Furthermore, the majority of these significantly upregulated genes (188/333, 56%) displayed low baseline expression (mean TPM <10 when grown in medium containing glucose). Among these significantly upregulated transcripts, 89 were encoded by PULs – a number significantly greater than expected by chance (*P* < 0.05, Fisher’s exact test). Thirty-six of these 89 genes encode CAZymes; they are annotated by PUL membership in Table 1*A* together with their predicted substrate specificities and their relative expression in PCDM-glucose versus -glucomannan. Table 1*B* also lists the five CAZymes involved in glycosidic bond cleavage whose expression was increased significantly by glucomannan but that are not located in PULs.

Transcription of all five CAZyme genes located in the BgF5_2 PUL 7 analog was significantly upregulated in the presence of glucomannan. Expression of the four other genes in this PUL, including those encoding components of the outer membrane SusC/SusD transporter complex and a gene (BBPDHENA_01821) homologous to a previously identified surface glycan-binding protein, was also significantly increased (Fig. 1*C*; ref. 36). Background expression of these PUL genes in medium containing glucose was low (mean TPM ranged from 0.4 to 255/gene) compared to the 1.5% glucomannan growth condition (mean TPM 60-1682; Dataset S3*A*). Upregulation of the outer-membrane proteins suggests a possible role in nutrient acquisition in vitro and, along with the additional upregulated components of PUL 7, a robust response of this PUL to glucomannan.

To explore the specificity of the PUL response to glucomannan, we determined the transcriptional response of each of the 22 PULs in the BgF5_2 genome. Of the 14 PULs composed of five or more genes, seven (including PUL 7) displayed significantly elevated expression in the presence of glucomannan (q < 0.05, GSEA; Fig. 1*D* and Dataset S3*B*). Using the transcriptional activity of the *SusC* and *SusD* homologs present in each PUL as markers of their expression, PUL 7 displayed the strongest response of all BgF5_2 PULs to growth in the presence of glucomannan (i.e., no other *SusC*/*SusD* pair displayed a log_2_ fold difference > 4; Dataset S3*A*).

UHPLC–QqQ-MS was performed to track consumption of glucomannan in *S. copri* BgF5_2-inoculated PCDM cultures harvested at mid-log phase (Dataset S4). Total mannose levels (both polysaccharide-bound and unbound), but not free mannose levels (not bound within polysaccharide), were approximately 100-fold lower in BgF5_2-inoculated spent medium relative to uninoculated controls (*P* < 0.05; Student’s *t* test; Fig. 1*E*), indicating consumption of the glucomannan polymer in inoculated cultures. Additionally, total glucose (and arabinose) levels were elevated (log_10_ fold difference = 1.02 and 0.77, *P* < 0.05) (Dataset S4*A*).

To further contextualize the transcriptional response of BgF5_2 to glucomannan, we expanded our expression analysis of metabolic pathways encoded by the BgF5_2 genome in mid-log phase cultures. A genome-wide functional annotation of *S. copri* BgF5_2 was generated previously using an approach based on the microbial community SEED database (mcSEED; 11, 37). This in silico metabolic reconstruction identified 158 intact metabolic pathways reflecting major nutrient biosynthetic and degradative capabilities (e.g., those involved in carbohydrate utilization, biosynthesis of amino acids and B vitamins). Expression of only one pathway, corresponding to “Tryptophan Biosynthesis,” was significantly altered (downregulated) by growth in the presence of glucomannan (NES: −1.97, q = 0.047; Dataset S3*C*).

### In Vitro Biochemical Characterization of PUL 7 and PUL 8 CAZymes.

**Fig. 2.**
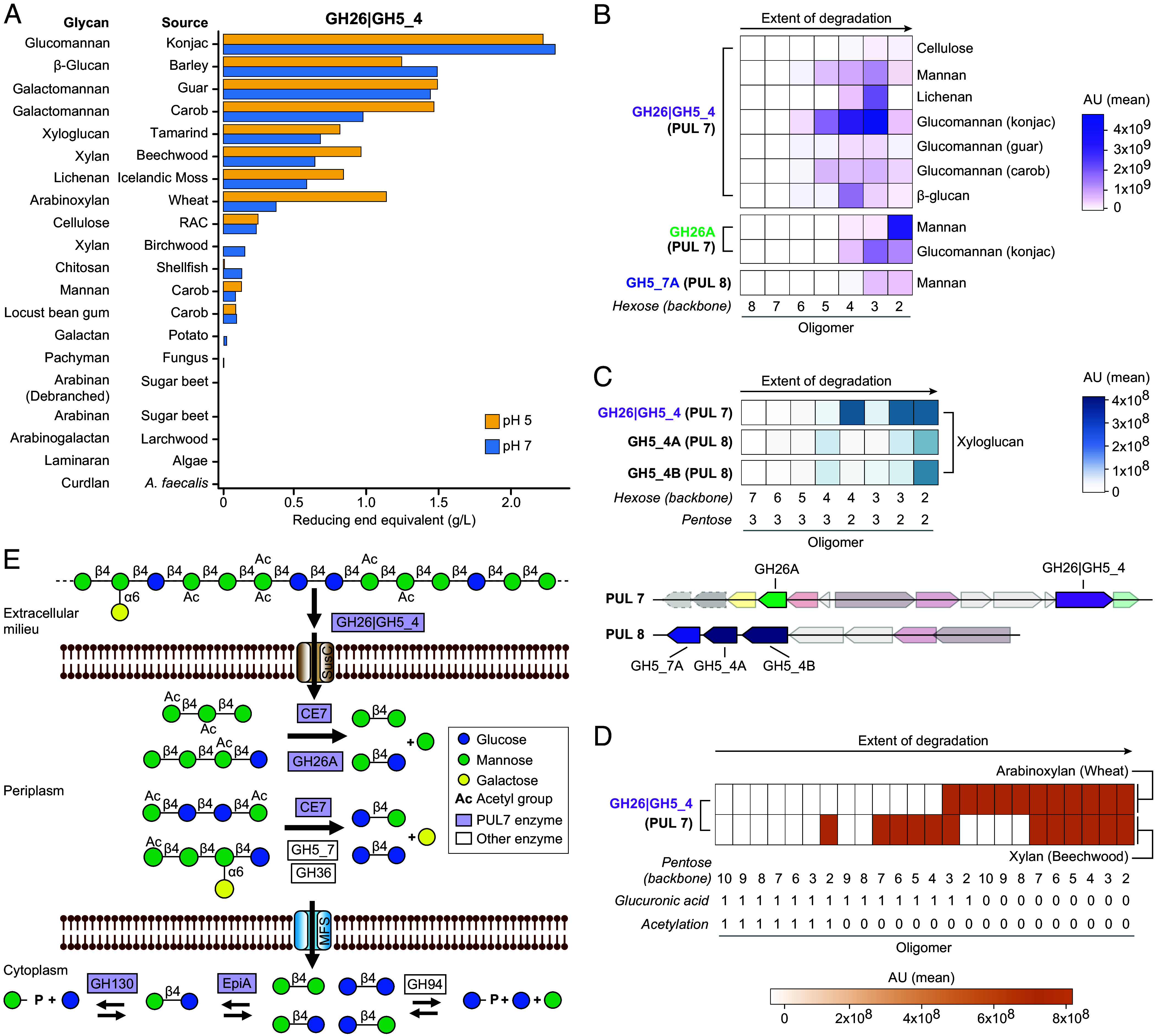
Biochemical characterization of enzymes encoded by PUL 7 and PUL 8. (*A*) Enzymatic activity of the bimodular GH26|GH5_4 CAZyme from PUL 7 against a range of polysaccharide substrates. Activity is measured as the change in concentration of reducing end equivalents (*SI Appendix*). (*B*–*D*) Heat maps of average signal intensities determined by LC-ESI-MS analysis of the products generated by PUL 7 and PUL 8 CAZymes after overnight reactions on (*B*) hexose-based substrates, including konjac glucomannan (GM-KON), carob galactomannan (GM-CAR), guar galactomannan (GM-GUA), β-1,4 mannan (MAN), barley β-glucan (BG-BAR), Regenerated Amorphous Cellulose (RAC), and lichenan (LIC-MOS), (*C*) xyloglucan substrate, specifically tamarind xyloglucan (XG-TAM) and (*D*) xylan backbone substrates, including wheat arabinoxylan (AX-WHE) and beechwood xylan (XYL-BEE). (*E*) Proposed schematic of complete glucomannan degradation in the cultured *S. copri* BgF5_2 isolate based on the biochemically confirmed activities of its PUL 7 and PUL 8 enzymes and their subcellular localizations as predicted by the presence and characteristics of signal peptides. Note that in panels *B*, *C* and *D*, the oligomer designations at bottom indicate the number of backbone hexose (*B* and *C*) or pentose (*C* and *D*) residues in each oligomer, along with whether glucuronic acid or acetylation modifications were detected (1, present; 0, absent). AU, arbitrary units.

We sought to confirm our homology-based activity predictions by validating in vitro the biochemical activities of MAG Bg0019 encoded PUL 7 CAZymes, as well as an adjacent open reading frame putatively annotated as an epimerase based on amino acid similarity. To determine the substrate specificities of the *S. copri* GH26|GH5_4 enzyme, its gene (locus tag MBINF3D6CCAE_04745 in MAG Bg0019, whose protein product is 93% identical to its BgF5_2 homolog; Dataset S1*A*) was codon-optimized, expressed in **Escherichia coli*,* purified via an added N-terminal 6× His tag (*SI Appendix*, Fig. S2) and incubated with each member of a panel of 20 polysaccharides (Fig. 2*A*). This screening assay, which measured release of reducing sugars (*SI Appendix*), revealed that 10 of the glycans were bona fide substrates (Fig. 2*A* and Dataset S5 *A* and *B*). *SI Appendix*, Fig. S3 describes the effect of pH on the enzyme’s activity using four of the active substrates possessing β-1,4 linkages, and its kinetic parameters when these substrates were assayed at the determined optimal pH.

The products of the reactions were subsequently analyzed using Liquid Chromatography-Electrospray Ionization-Mass Spectrometry (LC-ESI-MS; see *SI Appendix*). Among the 10 polysaccharides, GH26|GH5_4 exhibited the highest activity against glucomannan, producing the most oligomers (trimers, tetramers, and pentamers) (Fig. 2*B* and Dataset S6*A*). The enzyme also degraded the β-1,4-xylosidic linkages of xylans, primarily to hexamers for wheat arabinoxylan and tetramers for beech xylan (Fig. 2 *C* and *D* and Dataset S6*B*).

The levels of tri-, tetra-, and pentamers relative to other oligomers produced by the GH26|GH5_4 CAZyme suggests that it has an endolytic activity but requires other enzymes for complete substrate degradation. Typical patterns of PUL-mediated polysaccharide degradation suggest that the resulting glucomannan oligomers would be imported into the periplasm for further processing. Matrix-Assisted Laser Desorption Ionization Time-of-Flight (MALDI-TOF) mass spectra of reactions containing the products of GH26|GH5_4 treated glucomannan with and without added purified recombinant CE7 showed that the esterase can cleave the acetyl groups that occasionally decorate glucomannan (*SI Appendix*, Fig. S4).

The GH26A enzyme encoded by PUL 7 is predicted to be periplasmic. The reaction products generated by the purified GH26A enzyme (*SI Appendix*, Fig. S2) when incubated with mannan and glucomannan were also characterized using LC-ESI-MS (Dataset S6 *A* and *B*) and High-Performance Anion Exchange Chromatography with Pulsed Amperometric Detection (HPAEC-PAD; Dataset S6*C*). The results indicate that the oligomers resulting from mannan degradation are nearly all dimeric (88%), while those from glucomannan are largely dimeric (34%) or trimeric (50%) (Dataset S6*A*). These data suggest that GH26A contributes, along with GH26|GH5_4, to the breakdown of glucomannan, but is also unable to completely degrade this polysaccharide to its constituent monomers. We concluded that glucomannooligosaccharides resulting from extracellular GH26|GH5_4 activity are likely processed in the periplasm by GH26A to β-1,4-mannobiose and β-1,4-mannosylglucose and then transported to the cytoplasm.

The PUL 7 GH130 and adjacent epimerase (EpiA) enzymes are predicted to be cytoplasmic. Purified recombinant EpiA (*SI Appendix*, Fig. S2) was tested for activity on β-1,4 mannobiose, cellobiose, and mannosyl-β-1,4-glucose, and shown to convert each of these substrates to an equilibrium mixture of mannosyl-β-1,4-glucose, glucosyl-β-1,4-mannose and mannobiose by epimerization of the C2 position of the carbohydrate at the reducing end (*SI Appendix*, Fig. S5).Biochemical analysis of the purified recombinant GH130 enzyme showed that it is an inverting phosphorylase able to convert mannosyl-β-1,4-glucose to mannose-1-phosphate and glucose (*SI Appendix*, Fig. S6).

Only one protein encoded by PUL 7, namely GH26B, could not be assigned an enzymatic activity. Incubation of the recombinant purified protein with our panel of β-mannoside-containing substrates did not reveal any degradation. Binding assays, performed using affinity gels and nano-differential-scanning fluorimetry, with glucomannan, xyloglucan, galactomannan, mannan, and defined mannooligosaccharides (mannose to mannohexaose) all yielded negative results (see *SI Appendix*, including Dataset S5*B*). Although this protein shows clear sequence similarity to GH26 β-mannanases and β-mannosidases, it lacks the catalytic Glu residues characteristic of this family. We confirmed that it does not cleave β-mannosides. Because GH26B is predicted to be secreted to the external side of the outer membrane, we speculate that it may be operating as a binding protein, but the identity of its target is unknown.

MAG Bg0019 PUL 8 is positioned adjacent to PUL 7 and encodes a GH5_7 and two GH5_4 family members (Fig. 1*A*). Due to the close genomic proximity of the two PULs, we explored whether PUL 8 enzymes could also contribute to glucomannan degradation. The gene encoding the GH5_7 located within PUL 8 is the most proximal gene to PUL 7. The GH5_7 enzyme and *Cellvibrio mixtus* Man5B share 41% amino acid sequence identity and 57% similarity (40), leading us to postulate that it too is a β-mannosidase. Sequence homology suggests that the GH5_4 enzymes encoded by PUL 8 are likely β-1,4-glucosidases. We confirmed this latter prediction by expressing and purifying recombinant GH5_4A and GH5_4B followed by their incubation with tamarind xyloglucan (Dataset S6*B*). Together, these results agree with our finding that the gene encoding GH5_7 of PUL 8 is coinduced with PUL 7 proteins during growth on glucomannan (Fig. 1*C* and Dataset S3*A*).

**Fig. 3.**
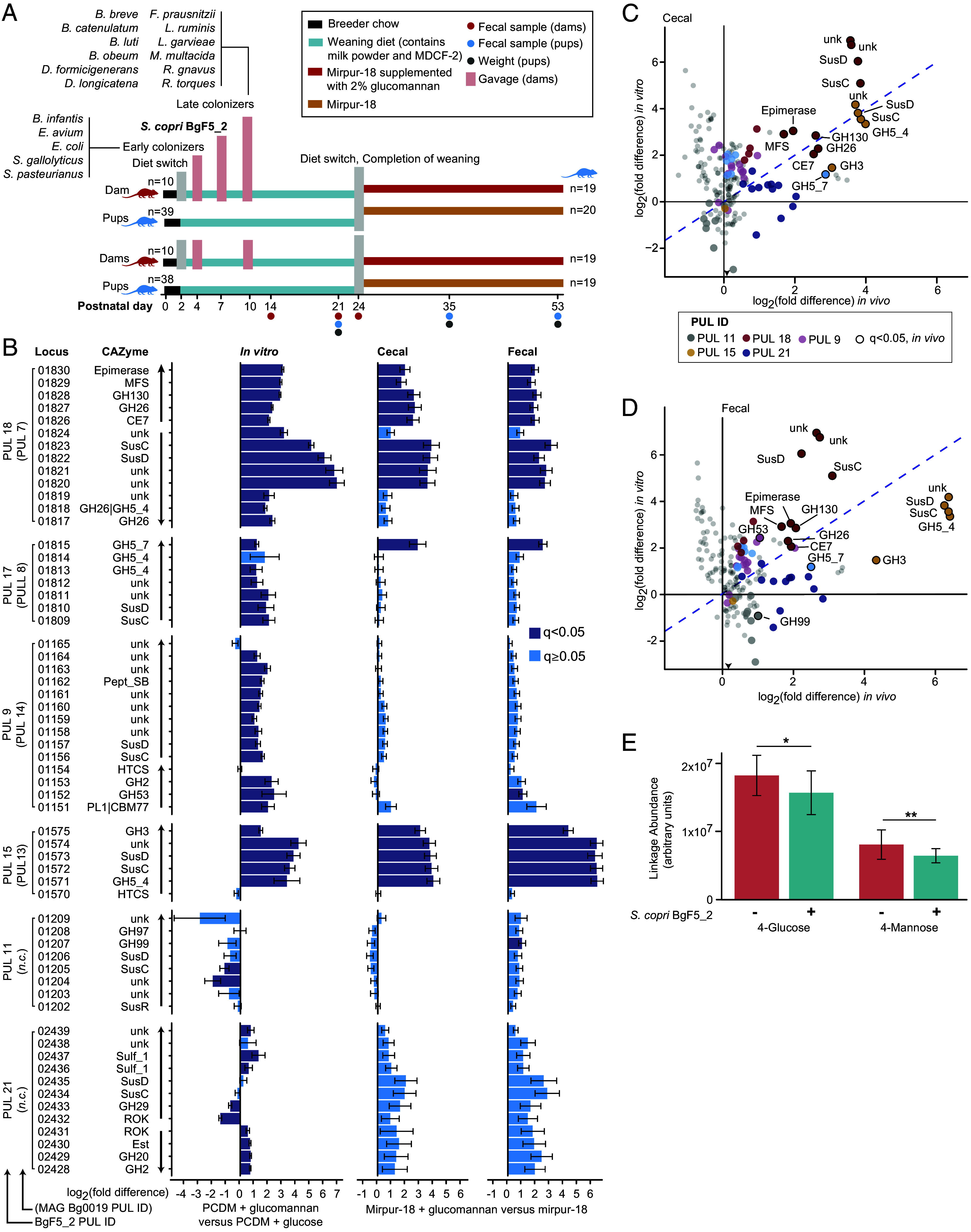
The transcriptional responses of *S. copri* BgF5_2 to dietary glucomannan in gnotobiotic mice. (*A*) Design of two independent gnotobiotic mouse experiments (n = 38 or 39 animals for each experiment) in which the defined microbial community, with or without *S. copri*, is transmitted intergenerationally from dams to pups. (*B*) Analysis of differential expression of genes in *S. copri* BgF5_2 PULs in vitro and in vivo. PUL genes and CAZyme identities are indicated at left, together with predicted polycistronic transcriptional units. PUL designations are indicated for the BgF5_2 isolate (leftmost PUL labels) or the MAG Bg0019 homologs (in parentheses) if present. Bars indicate differential expression as log_2_ fold differences between the glucomannan-supplemented and unsupplemented condition. Mean values and the SE are plotted. Statistical significance (q < 0.05, *MTXmodel*) is denoted by bar color. *n.c.*, not conserved in MAG Bg0019. (*C* and *D*) Correlation between the differential expression of PUL genes in vitro and in the cecum (*C*) or feces (*D*). PUL correspondence is indicated by color. Differential expression is described by log_2_ fold-difference as determined by DESeq2 (38) (in vitro, *y*-axis) or MTXmodel (39) (in vivo, *x*-axis). Statistical significance (q < 0.05) is indicated by black outlined points. (*E*) UHPLC–QqQ-MS quantification of 4-glucose and 4-mannose linkages in the cecal contents of mice colonized with or without *S. copri* BgF5_2.

As noted above, five glycan-cleaving enzymes located outside of PUL 7 and 8 were induced in vitro upon cultivation of *S. copri* BgF5_2 with glucomannan (Table 1*B*). One of these, BBPDHENA_00871, is 76% identical to a **Bacteroides* ovatus* galactomannan-specific α-galactosidase (41). Glucomannan preparations obtained from some plant sources such as Konjac can contain a fraction with galactose substituents (42). We speculate that this enzyme could remove the α-galactose side chains from galactoglucomannan side chains when they are present, thereby expanding the range of molecules processed by the CAZymes encoded by the PUL 7 and PUL 8 analogs present in the genome of strain BgF5_2. The predicted functions of the other induced enzymes could not be directly linked to the degradation of glucomannan; they may target as yet unknown glycans that are intimately associated to glucomannan in planta (see below). Fig. 3*E* summarizes the glucomannan degradation pathway inferred from our biochemical analysis of the enzymes encoded by *S. copri* MAG Bg00019 and its cultured representative BgF5_2.

### Assaying the Effects of Glucomannan in a Gnotobiotic Mouse Model of Maternal-Offspring Microbiota Transmission.

#### Experimental design.

We next examined the interaction between glucomannan, *S. copri* BgF5_2, and the host in vivo using a previously developed gnotobiotic mouse model that mimics the dynamics of postnatal microbiota development in Bangladeshi infants and children (16). In this model, a collection of genome-sequenced, age- and WLZ-associated gut bacterial strains cultured from Bangladeshi infants and children are introduced into germ-free dams. Following the experimental design outlined in Fig. 3*A*, a 5-member consortium of early phase bacterial colonizers of the infant intestine was administered to the dams on postpartum day 4, followed on postpartum day 10 by a 12-member consortium of organisms that normally colonize children during the period of complementary feeding (Dataset S7). *S. copri* was introduced by gavage after the early but before the late phase gavages. To explore the effects of glucomannan supplementation, pups were fed a diet sequence resembling that of infants and children living in the Mirpur urban slum where the MDCF-2 randomized controlled clinical trials had been performed. This consisted of an initial period of exclusive milk feeding (dam’s milk), followed by a weaning diet (“Mirpur-18”) that mimics that consumed by 18-mo-old Bangladeshi children living in Mirpur (16) supplemented with MDCF-2, and finally the Mirpur-18 diet with or without 2% w/w glucomannan on postnatal day 23 to 24 (P24 in the first experimental iteration, P23 in the second). The latter diets were administered for 4 wk prior to euthanasia (see *Methods*) on P53.

#### Effects on absolute abundances of community members.

We collected fecal samples at weaning (P23 or P24), P35 and P53, and cecal samples at P53 from a total 77 pups across the two experiments (n = 39 mice with and 38 without *S. copri* BgF5_2; Fig. 3*A* and Dataset S8). The absolute abundances of community members were defined by shotgun sequencing of DNA isolated from these biospecimens (*Methods*). At weaning, *S. copri* BgF5_2 had successfully colonized the intestines of all pups born to dams that had received this isolate (2.12 × 10^10^ ± 6.89 × 10^9^ genome equivalents, mean ± SD; Dataset S9 *A* and *B*).

After 10 d of consuming the glucomannan-supplemented Mirpur-18 diet (P35), there was a small but statistically significant increase in the fecal abundance of *S. copri* BgF5_2 that was not observed in mice consuming the unsupplemented diet (log_10_ fold-difference = 0.24 compared to weaning, *P* = 0.012 for the glucomannan-supplemented group; paired Mann–Whitney *U* test; Dataset S9 *A* and *B*). This increased abundance was sustained for the remainder of the experiment (log_10_ fold - difference = 0.24, *P* = 0.010; paired Mann–Whitney *U* test; Dataset S9 *A* and *B*). At euthanasia, fecal levels of *S. copri* were significantly higher in mice that consumed glucomannan-supplemented Mirpur-18 compared to those on Mirpur-18 alone (log_10_ fold-difference = 0.15; *P* = 4.39 × 10^−4^; Mann–Whitney *U* test; Dataset S9A). Only one other bacterial strain, **Streptococcus* pasteurianus,* displayed a significant difference (increase) in abundance between the experimental groups after 4 wk of glucomannan supplementation (log_10_ fold-difference = 0.37, *P* = 3.95 × 10^−3^; Mann–Whitney *U*; Dataset S9*B*).

#### Effects on microbial gene expression.

We performed microbial RNA-Seq on cecal contents and fecal samples collected at P53 to determine whether the glucomannan-induced genes we observed in vitro were also upregulated in vivo (*SI Appendix*). Our differential expression analysis strategy controlled for the important effect of varying *S. copri* abundance in the microbial community. The results across both experiments revealed that glucomannan supplementation produced significant differential expression of *S. copri* BgF5_2 genes in feces and cecal contents (Dataset S10 *A* and *B*). In total, 18 of the 42 genes upregulated with glucomannan supplementation in the cecum were encoded by PULs; all 18 were also significantly upregulated in feces.

The gene expression response of BgF5_2 to glucomannan in vivo, both at the PUL (Fig. 3*B*) and individual CAZyme (Fig. 3 *C* and *D*) levels, broadly recapitulated its response to this polysaccharide in vitro. When considered as multigene transcriptional units, nine PULs displayed significant up- or downregulation in the cecal contents or feces of mice consuming glucomannan-supplemented compared to unsupplemented diets (q < 0.05, GSEA; Dataset S10*C*). Importantly, the glucomannan-degrading PUL 7 analog was significantly upregulated by glucomannan in both intestinal compartments (q = 4.15 × 10^−6^ in the cecum; q = 2.05 × 10^−5^ in the feces); it was the most strongly upregulated PUL in the cecum, a primary site of microbial dietary polysaccharide degradation. When both mouse experiments were analyzed together, all nine of the genes comprising the PUL 7 analog were significantly upregulated in both the cecum and in feces (q < 0.05, MTXmodel, Fig. 3 *B*–*D* and Dataset S10 *A* and *B*). Two additional PULs displayed significant upregulation in both intestinal compartments: i) PUL 21, which has no equivalent in MAG Bg0019, is predicted to target Lewis antigens in mucins (q = 6.77 × 10^−6^ in the cecum, 2.23 × 10^−8^ in feces) and ii) PUL 15, which is equivalent to PUL 13 in MAG Bg0019, is predicted to degrade mixed-linkage β-1,3-glucans (q = 1.32 × 10^−8^ and q = 2.23 × 10^−8^, respectively) (Fig. 3*B*). The PUL 21 response and its predicted specificity for the Lewis antigens present in mucins suggests that strain BgF5_2 is able to perceive (and perhaps forage on) host carbohydrates.

Our finding that the bimodular enzyme encoded by the PUL 7 analog exhibits mixed linkage β-glucanase activity is consistent with the notion that its products would function as substrates for the predicted GH5_4 mixed linkage endoglucanase and the predicted GH3 β-glucosidase encoded by PUL 15. Only one CAZyme in PUL 8 (GH5_7 family member BBPDHENA_01815) exhibited a significant expression increase when mice consumed glucomannan; this enzyme is a β-mannosidase predicted to be secreted to the periplasm (Table 1*A* and Dataset S10 *A* and *B*).

Several non-PUL CAZymes in *S. copri* BgF5_2 also displayed significant differential expression in response to glucomannan. In both the cecum and feces, CAZymes belonging to the GH94 family (BBPDHENA_00871) and the GH36 family (BBPDHENA_00871) were upregulated by glucomannan (Dataset S10 *A* and *B*). Interestingly, the same GH36 CAZyme, a predicted α-galactosidase, was also significantly upregulated in response to glucomannan supplementation in vitro. This is consistent with the occurrence of α-galactose side chains in plant glucomannans. Finally, since no β-glucosidase is present in the BgF5_2 genome, and GH130 is specific for disaccharides with a mannose on the nonreducing end, it is reasonable to speculate that the GH94 enzyme, which is 67% identical to a cellobiose phosphorylase from *Saccharophagus degradans* (43), could be involved in the cytoplasmic breakdown of cellobiose and glucose-1,4-mannose arising from glucomannan degradation.

We extended our microbial RNA-Seq analysis beyond *S. copri* to other microbial community members to determine whether expression of CAZymes in other bacteria were altered in response to glucomannan supplementation, and whether any observed changes were dependent on the presence of *S. copri* in the community. Dataset S11 lists the CAZymes encoded in the genomes of each member of the defined bacterial community that colonized mice, their changes in expression in the cecal contents and fecal biospecimens as a function of the presence or absence of glucomannan, as well as the *S. copri* dependence of these transcriptional responses to glucomannan. When *S. copri* was present, a total of eight CAZyme genes were significantly differentially expressed in the cecum and 28 in the feces; all these genes were upregulated with 6 of the 8 cecal and 5 of the 28 fecal CAZyme genes displaying *S. copri*-dependent responses to glucomannan. The two CAZymes exhibiting significant glucomannan- and *S. copri*-dependent responses in both cecal and fecal communities are members of families GH36 (predicted α-galactosidase) and GH1 (predicted β-galactosidase, β-glucosidase and 6-phospho-β-glucosidase activities); both are from *S. pasteurianus*. However, with two exceptions, none of the CAZymes whose expression was significantly altered by glucomannan have predicted secretion signals, including the only predicted GH2_13 β-mannosidase (from *Bifidobacterium catenulatum*). The two exceptions were GH23 and GH73 enzymes from *S. pasteurianus* that are predicted to degrade bacterial cell surface peptidoglycans. Inspection of the genomes of additional microbial community members failed to disclose any other CAZymes with the potential to degrade glucomannan that displayed both significant differential expression and a secretion signal peptide.

We complemented our transcriptional analyses with UHPLC-QqQ-MS analyses of glycans to quantify degradation by *S. copri* and other microbes in vivo. The cecums of mice colonized with the BgF5_2 strain whose diet had been supplemented with glucomannan contained significantly lower levels of β-1,4-glucosidic and β-1,4-mannosidic linkages compared to animals fed the same diet but not colonized with *S. copri* (*P* < 0.05, Mann–Whitney *U* test; Fig. 3*E* and Dataset S12).

Together, our in vitro and gnotobiotic mouse studies show that glucomannan, a prebiotic glycan present in MDCF-2, can be directly metabolized by *S. copri* BgF5_2. The genome of this isolate is highly similar—including its glucomannan-targeting PUL repertoire—to growth-associated *S. copri* MAGs identified in fecal samples from two randomized controlled trials of MDCF-2 in different cohorts of Bangladeshi children. Our transcriptional and biochemical analyses indicate that *S. copri* acts as a principal metabolizer of glucomannan in vitro and in vivo though the action of a key multifunctional bimodular GH26|GH5_4 CAZyme and other colocalized enzymes (summarized in Fig. 2*E*). Further, these analyses suggest the possibility that this enzyme initiates similar mechanisms of polysaccharide degradation relevant to additional putative bioactive glycans in MDCF-2 (*SI Appendix*, Fig. S7).

We have previously used this gnotobiotic model to demonstrate an MDCF-2- and *S. copri*-dependent enhancement of body weight compared to that observed in animals harboring a defined community without this organism (16). In the current study, mice colonized with *S. copri* BgF5_2 and consuming the Mirpur-18 diet supplemented with glucomannan did not exhibit increased body weights across the time course of the experiment or in cross-sectional analyses at P35 or P53 (linear models Eq. 1 and Eq. 2; see Dataset S8), suggesting that additional components of MDCF-2 are required to induce this phenotype.

## Discussion

This report describes an approach for advancing results from randomized clinical trials (RCTs) that had documented the superior effects of a MDCF-2 compared to a standard RUSF in improving ponderal and linear growth in Bangladeshi children with MAM, to a search for affordable, sustainable sources of bioactive glycans identified in MDCF-2 that could, in the future, be used as prebiotics to promote healthy microbiome functions. The present study highlights the advantage of extending beyond in silico predictions to also employ biochemical methods to directly document the substrate specificities of carbohydrate-active enzymes (CAZymes) present in microbial therapeutic targets in order to rationally select prebiotic candidates.

Food is rarely composed of a single polysaccharide, and in plants glucomannan, arabinoxylan, xyloglucan, and mixed linkage β-glucan are often presented as intimate mixtures. The bioactive glycans identified in MDCF-2 encompass these structures. As noted *above*, we have previously used culture-independent methods to document that growth recovery produced by MDCF-2 treatment of undernourished Bangladeshi children was associated with strain level variation in *S. copri*. In 12 to 18-mo-old children with primary MAM living in the Mirpur urban slum district of Dhaka, Bangladesh, two *S. copri* MAGs with statistically significant positive associations with weight gain (Bg0018 and Bg0019) shared PULs that were not present in other *S. copri* MAGs present in the study population (11). The predicted substrates of the majority (5/7) of PULs that were highly conserved between these two MAGs were glycans present in MDCF-2, including mannans and galactans represented at significantly higher levels in this therapeutic food compared to RUSF. A second RCT of similarly aged children living in urban and rural sites in Bangladesh who had been treated for post-SAM MAM with MDCF-2, disclosed an additional growth-associated *S. copri* MAG that shared a strikingly similar PUL conservation and predicted substrate profile with Bg0019 (9). We were able culture a *S. copri* strain (BgF5_2) that captured the PULs present in these growth-associated MAGs, including a conserved homolog of MAG Bg0019’s PUL 7 that encodes a bimodular GH26|GH5_4 CAZyme; this enzyme is predicted to hydrolyze both β-1,4-mannosidic and β-1,4-glucosidic linkages and is largely restricted to *S. copri.* The current study demonstrates that *S. copri* BgF5_2 can utilize glucomannan as an energy source in vitro. In vitro biochemical analyses of the activities of purified CAZymes encoded by PUL 7 and 8 and the transcriptional responses of their genes to exposure to glucomannan in vitro and in vivo indicated that expression of this bimodular CAZyme is inducible by glucomannan and that it is able to cleave glucomannan as well as xyloglucan, arabinoxylan, and mixed linkage β-glucan.

Fig. 2*E* and *SI Appendix*, Fig. S7 show versions of a working model, based on our results, where the bimodular CAZyme functions as a multisubstrate “sentinel” that provides a variety of oligosaccharides from a variety of β-linked glycans, with each oligosaccharide type able to induce different PULs and non-PUL enzymes depending on the glycan composition in complex natural diets. This view is supported by the fact that for each of the substrates of GH26|GH5_4 examined (glucomannan, arabinoxylan, xyloglucan, and mixed linkage β-glucan) there is a least one suitable PUL plus non-PUL CAZymes for complete processing in the periplasm and the cytoplasm (Fig. 2*E* and *SI Appendix*, Fig. S7). A corollary is that strain-level variation in the CAZyme repertoires of *S. copri* and other growth-associated taxa in a given microbial community will be a key determinant of their responsiveness to MDCF-2 glycans and the oligosaccharide products of their CAZyme-mediated degradation.

In a previous report (16), we used the same dam-to-pup microbiota transmission model described in the present study to show that *S. copri* BgF5_2 produced an MDCF-2-dependent effect on growth. When animals were fed MDCF-2, weight gain was significantly greater in mice colonized with *S. copri* compared to those lacking it; this was not the case when they were fed a “Mirpur” diet representative of that typically consumed by 12- to 18-mo-old children living in the Mirpur slum. In the present study, glucomannan supplementation of this Mirpur diet administered to gnotobiotic mice colonized with other growth-discriminatory strains did not produce a significant *S. copri*-dependent weight gain response. We have yet to determine whether the growth response observed in our mouse model (or in the children that this model is designed to represent) is directly due to the products of degradation of MDCF-2 glycans by *S. copri* PUL 7/8 CAZymes, or due to other metabolic pathways whose expression is regulated by exposure to these substrates/products in *S. copri* or other community members. We have not yet successfully developed the means to genetically manipulate *S. copri* BgF5_2 PULs to directly determine the role of the bimodular enzyme in glucomannan and MDCF-2 metabolism, and in host responses. Nonetheless, the fact that a consistent, statistically significant weight phenotype was not produced in gnotobiotic mice supplemented with glucomannan alone suggests that additional glycans present in MDCF-2 will likely need to be combined to produce the benefits observed in our gnotobiotic mouse model and in participants enrolled in both the primary MAM and post-SAM MAM clinical trials. These effects not only include ponderal and linear growth improvements, but also a multiplicity of physiologic changes revealed by monitoring changes in the plasma proteome; the latter include biomarkers and mediators of musculoskeletal and CNS development and regulators of metabolic and immune functions (9, 10, 44).

Although glucomannan was not sufficient to produce a ponderal growth phenotype in gnotobiotic mice when added to a Mirpur diet, it may be valuable as an inducer of the bimodular enzyme in *S. copri* strains that possess this gene. Induction of this multifunctional enzyme would offer the opportunity to promote its utilization of a mixture of other prebiotic glycans that act as its substrates in our in vitro biochemical assays. Testing this hypothesis is a logical next step in a prebiotic discovery effort, as would be the development of a qPCR assay for the gene encoding this bimodular enzyme in order to determine whether its presence correlates with host responsiveness to MDCF-2.

## Methods

### Genome Sequencing and annotation.

The *S. copri* genome sequences and their annotation, including those for cultured strain BgF5_2 and MAG Bg0019 have been published previously (11, 16) and are detailed in Dataset S2. CAZyme, PUL, and mcSEED annotations were performed according to methods described in earlier publications (11, 16, 37) and in *SI Appendix*. Subcellular/extracellular CAZyme localization was predicted using SignalP (v6.0) (45). Details of phylogenetic reconstruction and analysis of the distribution of the bimodular GH26|GH5_4 can be found in *SI Appendix*.

### Growth and Microbial RNA-Seq Analysis of *S. copri* BgF5_2 in Glucomannan-Supplemented Cultures.

All routine culture and carbohydrate screening studies employed *S. copri* defined medium (PCDM) prepared as previously described (35) with minor modifications (11). PCDM was supplemented with glucomannan or glucose as described in *SI Appendix*. Bacterial growth was quantified in triplicate cultures using optical density measurements at 600 nm (OD_600_).

For RNA-Seq studies, *S. copri* was grown in PCDM supplemented with 1% glucose or 1.5% glucomannan. At mid-log phase (OD_600_ 0.4 to 0.5), aliquots were withdrawn from the monocultures and frozen at −80 °C. Total nucleic acids were subsequently extracted, and cDNA libraries were prepared and sequenced (Illumina NovaSeq S4 flow cell) as described in *SI Appendix*. The resulting reads were subjected to routine quality control, mapped to the *S. copri* BgF5_2 genome using kallisto (46), and differential expression was determined for individual genes (DESeq2, ref. 38) or gene sets (fgsea, ref. 47).

### Quantification of Monosaccharides and Linkages.

Monosaccharide analyses of frozen samples of in vitro culture supernatants, freeze-dried glucomannan powder, animal diets, or cecal contents were adapted from previous publications (23, 48). Methods for quantification of glycosidic linkages in the same sample types were from previous publications with modifications (22, 49). See *SI Appendix* for additional details.

### Biochemical Assays of Recombinant Enzymes.

Genes selected for characterization of the biochemical activities of their protein products were initially codon-optimized for expression in *E. coli*, an N-terminal 6×His tag was added and then subcloned into appropriate expression vectors. Recombinant enzymes were produced in *E. coli* BL21 (DE3) cells under antibiotic selection; proteins were extracted and purified using Ni resin and size exclusion chromatography (*SI Appendix*).

After pH optimization on known substrates, activity assays for glycoside hydrolases, esterases, epimerases, and phosphorylases were performed in triplicate using 5 g/L of each test substrate (1 g/L for the epimerase/phosphorylase) in 50 mM buffer UB4 (*SI Appendix*). Reactions were incubated at 37 °C and halted using the DNS method (50). Absorbance at 540 nm and a standard glucose curve was used to determine reducing sugar content where appropriate. Reaction products were characterized using mass spectrometry or HPAEC-PAD as described in *SI Appendix*. For the catalytically inactive GH26B enzyme, binding assays were performed using native polyacrylamide affinity gel electrophoresis as described previously (51).

### Mouse Experiments.

Experiments involving gnotobiotic mice were performed using protocols approved by the Washington University Animal Studies Committee. Germ-free C57BL/6J mice were maintained in plastic flexible film isolators (Class Biologically Clean Ltd) at 23 °C with a 12-h light cycle. Mice were mated, became pregnant, and were subjected to a diet and microbial colonization protocol as described in Fig. 3*A* and *SI Appendix*. Glucomannan was administered through the drinking water (Experiment 1) or via direct incorporation into the Mirpur-18 diet (Experiment 2). Collection of feces over the course of the experiment, plus the collection of additional intestinal biospecimens and other tissues is described in *SI Appendix*. Details of weight analysis are described in *SI Appendix*. In accordance with the Washington University in St. Louis Animal Euthanasia Policy (https://research.washu.edu/animal-euthanasia-policy/), animals were euthanized by cervical dislocation by trained personnel to prevent possible effects of asphyxiation with CO_2_ on intestinal physiology. The effect of glucomannan on bacterial strain abundances was determined by shotgun sequencing of microbial community DNA using spike-in standards, an Illumina NextSeq instrument and previously published methods (16). DNA reads were processed using COPRO-Seq (52) and adjusted to yield absolute bacterial abundances, which were log-transformed and analyzed with Mann–Whitney tests and Benjamini–Hochberg correction for false discovery rate.

Bacterial transcriptional profiling was performed on fecal and cecal biospecimens collected as above. Briefly, cDNA libraries were generated from isolated RNA using the Illumina “Total RNA Prep with Ribo-Zero Plus” kit and sequenced on the Illumina NovaSeq platform. Resulting reads were trimmed, filtered and mapped to the genomes of all colonizing bacterial strains using kallisto (46). Differential expression of mRNA transcripts was analyzed using MTXmodel (39) so that false positive differential expression due to differences in bacterial abundances could be controlled. Transcripts were deemed significantly differentially expressed (that is, had “diet” coefficients that were significantly different from 0) if their Benjamini–Hochberg adjusted *P*-value was less than 0.1. See *SI Appendix* for additional details.

## Supplementary Material

Appendix 01 (PDF)

Dataset S01 (XLSX)

Dataset S02 (XLSX)

Dataset S03 (XLSX)

Dataset S04 (XLSX)

Dataset S05 (XLSX)

Dataset S06 (XLSX)

Dataset S07 (XLSX)

Dataset S08 (XLSX)

Dataset S09 (XLSX)

Dataset S10 (XLSX)

Dataset S11 (XLSX)

Dataset S12 (XLSX)

Dataset S13 (XLSX)

## Data Availability

Microbial community DNA shotgun sequencing datasets and microbial RNA-seq datasets have been deposited in SRA (Accession: PRJNA1124109; ref. 53). UHPLC-QqQ-MS and LC-ESI-MS datasets are available in Glycopost (GPST000497) (54). Analytical code has been deposited at GitLab (https://gitlab.com/Gordon_Lab/zhou_hibberd_et_al_2025/) and accessioned at Zenodo (55).
