# Supplementary material for: Glycoside hydrolase–mediated glucomannan catabolism in Segatella copri, a target of microbiota-directed foods for malnourished children
Source: Proc Natl Acad Sci U S A. 2025 Dec 2;122(49):e2521522122. doi: 10.1073/pnas.2521522122 (PMC12704710; doi:10.1073/pnas.2521522122)
Supplement: Supplementary file 1 — Appendix 01 (PDF) [file pnas.2521522122.sapp.pdf]

Supporting Information for:

Glycoside hydrolase-mediated glucomannan catabolism in *Segatella copri*, a target of microbiota-directed foods for malnourished children

Cyrus Zhou, Matthew C. Hibberd, Evan M. Lee, Bo Pilgaard, Marlene Vuillemin, Emma Kiehn, Suzanne Henrissat, Marie A. Crane, Jiye Cheng, Lara Pfaff, Anne S. Meyer, Jesper Holck, Nicolas Terrapon, Juan J. Castillo, Garret Couture, Carlito B. Lebrilla, Dmitry A. Rodionov, Michael J. Barratt, Bernard Henrissat and Jeffrey I. Gordon

This PDF file includes:

Supporting Text
Figures S1 to S7
Legends for Datasets S1 to S13
SI References

Other supporting materials for this manuscript include the following:

Datasets S1 to S13

SUPPORTING TEXT

SUPPLEMENTAL METHODS

Genome sequencing and annotation

The genome sequence for the *S. copri* type strain DSM 18205 is publicly available (https://www.ncbi.nlm.nih.gov/datasets/taxonomy/537011/), and those for the cultured Bangladeshi BgF5_2 strain and the Bg0019 MAG have been published (1, 2). CAZymes and PULs were identified in these genomes using methods described in earlier publications (1, 2). For PUL 7 proteins without annotations in the CAZy database, sequence comparison to known surface glycan-binding proteins was performed using the InterProScan tool and reference sequences contained in the InterPro database (3). *In silico* phenotype predictions and gene-level annotations using the mcSEED database were generated as reported previously (1, 2, 4) using an updated database able to predict 158 metabolic pathways.

For phylogenetic analysis, publicly available representative genomes of a diversity of Prevotellaceae were downloaded from the BV-BRC repository, along with all available "good" (per BV-BRC criteria) quality representatives of *Segatella copri* (5). Additional representative genomes were obtained from the National Center for Biotechnology Information Genome database (6) and from isolates we obtained from the feces or duodenal aspirates of Bangladeshi participants in our randomized controlled trials (RCTs, refs. 1, 2, 4). The identities and details for all MAG and isolate genomes are described in **Dataset S2**. Each genome, including the *Bacteroides thetaiotaomicron* VPI-5482 genome as an outgroup, was processed using the checkm (7) 'lineage_wf' command to obtain aligned, concatenated sets of 43 universal, single copy marker genes. A phylogenetic tree was generated from these marker gene alignments using fasttree (v2.1.10, ref. 8) based on the Jones-Taylor-Thornton model and 'CAT' evolution rate, which was then rescaled using the Gamma20 optimization. The tree was rooted on the *B. thetaiotaomicron* genome, and nodes were collapsed (9) based on topology and/or genome taxonomy (10) to enable visualization in R using ggtree (v3.14.0, ref. 11).

Analysis of the distribution of the MAG Bg0019 encoded GH26|GH5_4 enzyme among additional Bacteroidota genomes proceeded in two steps. Our initial analysis encompassed 1889 genomes in the CAZy database (12). A module-based search was used to determine the presence/absence of this multifunctional enzyme in these genomes. Our secondary analysis considered a partially overlapping set of 911 genomes focused on *Segatella* species. A local BLAST (13) database was generated from the contig set corresponding to each genome. Using the Bg0019 GH26|GH5_4 reference amino acid query, a TBLASTN search was performed against the *Segatella* genome database. Tabular results were filtered to include only near full-length or full-length alignments (≥700 of the 786 amino acids comprising the bimodular enzyme) and aligned to the reference tree using ggtree in R (11).

Growth of *S. copri* BgF5_2 in carbohydrate-deficient defined medium

We previously reported the use of a *S. copri* defined medium (PCDM) that does not support growth unless the medium is supplemented with additional carbon sources (1). PCDM was modified from a previously published recipe (14) with the addition of 1 mL of a stock solution containing 1.9 mM hematin (Sigma, Cat # H3281) and 0.2 M L-histidine (Sigma, Cat # H6034) to 1 L of medium.

Purified glucomannan derived from Konjac (Megazyme, P-GLCML) or glucose (Sigma, Cat # G8270) was dissolved into purified water (Sigma-Aldrich, Milli-Q® IQ 7000, ZIQ7000T0C) to form either a 3% or 2% w/v solution, respectively. The 3% glucomannan solution was sterilized by autoclaving at 121 °C for 15 minutes at 15 psi. The 2% glucose solution was filter-sterilized using 0.22 μm filters (Millipore; Stericup, SKU S2HVU02RE). Both solutions were allowed to equilibrate under anaerobic conditions for ~3 days prior to use. The 3% glucomannan solution and 2% glucose solution were mixed separately with PCDM to create PCDM with 1.5% glucomannan or 1% glucose, respectively. The sterility of these solutions was determined by plating 10 μL aliquots of each onto Brain Heart Infusion (BHI) agar (Difco 241380) supplemented with 10% horse blood. Absence of visible colonies after 3 days indicated the sterility of growth media.

The BgF5_2 *S. copri* strain was isolated previously (1). 100 μL of freezer stocks were inoculated into 5 mL of Wilkins-Chalgren (WC) medium (Oxoid, Cat # CM0643) and grown at 37 °C under anaerobic conditions (atmosphere; 75% N_2, 20% CO_2, 5% H_2) in a Coy chamber (Coy Laboratory Products, Grass Lake, MI) without shaking for approximately 16 hours until reaching stationary phase. A 5 μL inoculum of this culture was added to individual wells of a 96-well plate (TPP, Cat # 92096) containing 195 μL of PCDM with 1% glucose, PCDM with 1.5% glucomannan, or unsupplemented PCDM (1:40 dilution). The assay plate was sealed with optically clear film (Axygen, Cat # UC-500) and loaded into a BioTek Eon microplate spectrophotometer inside the anaerobic chamber. The spectrophotometer measured the OD_{600} of each well every 15 minutes for 96 hours at 37 °C. Prior to each measurement, each plate was agitated for five seconds. Readings were exported in tabular form and analyzed in R using custom scripts to determine maximum OD_{600} and growth rate. OD_{600} measurements across all wells were normalized to the lowest OD_{600} observed in wells containing unsupplemented PCDM. Successful growth on glucomannan as the sole carbon source was determined based on observing $OD_{600} > 0.3$ at or after the 24 hr timepoint. All growth curves were performed in triplicate.

Samples for transcriptional profiling and mass spectrometric analysis were collected from *S. copri* BgF5_2 grown in PCDM supplemented with either 1% glucose or 1.5% glucomannan. *S. copri* BgF5_2 cultures were grown in 14 mL round bottom tubes (Corning, Cat # CLS352051) at 37 °C under anaerobic conditions. A 100 uL aliquot was taken every 3 hours to determine the OD_{600}. When samples reached an OD_{600} between 0.4 - 0.5, tubes were removed from incubation and centrifuged at 5000 x g at 4 °C for 5 min. Samples designated for microbial RNA-Seq had their supernatants decanted and the remaining pellets were flash frozen in liquid nitrogen prior to storage at -80C. For samples designated for mass spectrometry, both supernatant and pellets were flash frozen (separately) and stored at -80 °C.

Microbial RNA-Seq of *S. copri* BgF5_2 grown in PCDM with or without glucomannan

Cryopreserved cell pellets from cultures grown to log phase (OD_{600} 0.4-0.5) were thawed, nucleic acids were extracted, and cDNA libraries were generated using the Illumina Stranded Total RNA Prep Ligation with Ribo-Zero Plus kit following the manufacturer's recommended protocol. Libraries were balanced, pooled, and sequenced using an Illumina NovaSeq instrument and S4 flow cells ($9.91 \times 10^5 \pm 6.54 \times 10^4$ 150 nt paired-end reads/sample). Reads were trimmed using *trimgalore* (v0.6.4, ref. 15) to remove low quality bases and adapter sequences and trimmed read pairs in which one or both reads was less than 100 nt in length were filtered out. Filtered reads were mapped onto the *S. copri* BgF5_2 genome using *kallisto* (v0.43.0, ref. 16).

Differential abundance comparisons were performed on gene counts between the glucomannan-supplemented conditions and the glucose-supplemented reference cultures in R (v4.1.2) using *DESeq2* (v1.34.0, ref. 17). Genes that had a false-discovery rate (FDR) corrected P-value (Benjamini-Hochberg method) <0.05 and a $\log_2$ fold difference ≥1 were considered significant. Annotations to which genes in the *S. copri* BgF5_2 genome were part of PULs and mcSEED functional pathways can be found in **Dataset S3** and in a previous publication (2).

Gene set enrichment analysis (GSEA) was performed using the R package *fgsea* (v1.20.0, ref. 18) on genes that were ranked by their $\log_2$-fold differences (genes enriched by glucose were arbitrarily given negative $\log_2$-fold differences; gene enriched by glucomannan were given positive $\log_2$-fold differences). This set of analyses allowed us to identify differentially expressed metabolic pathways comprised of ≥3 genes between our two media conditions *in vitro* or diet conditions *in vivo*. Enrichment results were considered statistically significant if they had FDR-corrected *P*-values < 0.05 (Benjamini-Hochberg method).

Quantification of monosaccharides and linkages

Frozen samples of bacterial culture supernatant, freeze-dried glucomannan powder, animal diets or cecal contents were pulverized to a fine powder using 2 mm stainless steel beads (for foods) or 2 mm glass beads (for cecal contents). A 10 mg/mL stock solution of each sample was prepared in Nanopure water (Thermo Fisher). All stock solutions were again bead homogenized, incubated at 100 °C for 1 h, bead homogenized again, and stored at −20 °C until further analysis. For quantification of monosaccharides, methods were adapted from previous publications (19, 20). Each sample was subjected to acid hydrolysis (4 M trifluoroacetic acid for 1 h at 121 °C) followed by addition of 855 µL of ice-cold Nanopure water. Hydrolyzed samples, plus an external calibration standard comprised of 14 monosaccharides with known concentrations (0.001–100 µg/mL each) were derivatized with 0.2 M 1-phenyl-3-methyl-5-pyrazolone (PMP) in methanol plus 28% NH_4OH for 30 minutes at 70 °C. The derivatized glycosides were fully dried by vacuum centrifugation, reconstituted in Nanopure water, and excess PMP was extracted with chloroform. A 1 µL aliquot of the aqueous layer was injected into an Agilent 1290 Infinity II ultrahigh performance liquid chromatography (UHPLC) system, analytes were separated using a 2-minute isocratic elution on a C18 column (Poroshell HPH, 2.1 × 50 mm, 1.9 µm particle size, Agilent Technologies) and analyzed using an Agilent 6495A triple quadrupole mass spectrometer (QqQ-MS) operated in dynamic multiple reaction monitoring (dMRM) mode. Monosaccharides were identified and quantified by comparison to the external calibration curve.

Methods for quantification of glycosidic linkages were adapted from a previous publication with modifications (21, 22). Under an argon atmosphere, a 5 µL aliquot from each homogenized stock solution of a sample was permethylated in a 200 µL reaction that contained 5 µL saturated NaOH and 40 µL iodomethane in 150 µL of DMSO. Permethylated glycosides were extracted with dichloromethane, and the extract was dried by vacuum centrifugation. The extracted glycosides were subjected to acid hydrolysis (4 M trifluoroacetic acid for 2 hr at 100 °C) followed by vacuum centrifugation to dryness. Samples were then derivatized with PMP as described above for the monosaccharide analysis, followed by another vacuum centrifugation to complete dryness. Methylated monosaccharides were then dissolved in 100 µL of 70% methanol in water. A 1 µL aliquot of the aqueous layer was injected into an Agilent 1290 Infinity II UHPLC system, separated using a 16-minute gradient elution on a C18 column (ZORBAX RRHD Eclipse Plus, 2.1 × 150 mm, 1.8 µm particle size, Agilent Technologies), and analyzed using an Agilent 6495A QqQ-MS operated in multiple reaction monitoring (MRM) mode. A

pool of oligosaccharide standards and a reference MRM library were used to identify and quantify glycosidic linkages in all samples.

Biochemical assays of recombinant enzymes

Plasmids - All genes were codon-optimized for *E. coli* expression and encoded signal peptides, when present, were removed. The genes encoding GH26|GH5_4, GH26A, GH26B, CE7 and EpiA were synthesized by Twist Bioscience (San Francisco, USA) and cloned into a pHTP1 vector (NZYTech, Lisbon, Portugal), incorporating a N-terminal 6×His tag and a Tobacco Etch Virus (TEV) cleavage site. The GH130 gene was synthesized by GenScript (Piscataway, USA) and cloned into the pET22b+ vector with an N-terminal 6×His tag.

Production and purification of recombinant proteins - Recombinant enzyme production was performed in *E. coli* BL21 (DE3) cells. Cultures were grown from a preculture in 1 L of Luria Broth (LB) supplemented with the appropriate antibiotic at 37 °C. Cells were cultured until OD_{600} of 0.6–0.8 was reached, at which point protein expression was induced with 0.5 mM IPTG. Cultures were incubated for 20 hours at 18 °C before cells were harvested by centrifugation for 15 minutes at 4 °C. Cell pellets were resuspended in binding buffer (20 mM sodium phosphate, 500 mM NaCl, and 50 mM imidazole, pH 7.4) and lysed on ice using a Q700 Sonicator equipped with a Microtip Probe 4420 (Qsonica, Newtown, CT, USA). Cell debris were removed by centrifugation at 4 °C, and the supernatant was filtered before purification. Proteins were purified using a 2 mL Sepharose Ni resin column (Cytiva, Marlborough, USA). Proteins binding non-specifically were removed by washing with binding buffer, and His-tagged proteins were eluted using elution buffer (250 mM imidazole, 20 mM sodium phosphate, 500 mM NaCl, pH 7.4). Further purification was performed by size exclusion chromatography using a Sephadex 60/100 column in running buffer (20 mM sodium phosphate, 100 mM NaCl, pH 7.4). Protein concentrations were determined using a NanoDrop spectrophotometer (NanoLite, Wilmington, USA) and extinction coefficients were predicted by Benchling (San Francisco, USA). Proteins were visualized by SDS-PAGE using Mini-PROTEAN gels (Bio-Rad, Hercules, USA).

Activity screening of glycoside hydrolases – Assays were performed in triplicate in 200 µL reactions that contained 1 µM purified enzyme, 50 mM buffer UB4 (23) pH 5 or pH 7, and 5 g/L substrate. Ivory nut mannan, barley mixed-linkage β-glucan, beechwood xylan, wheat arabinoxylan, konjac glucomannan, Icelandic moss lichenan, carob galactomannan, guar galactomannan, tamarind xyloglucan, potato galactan, larchwood arabinogalactan, carboxymethyl curdlan, carboxymethyl pachyman, cellobiose, mannobiose and mannotriose were purchased from Megazyme (Bray, Ireland). Laminarin and chitosan were obtained from TCI (Tokyo, Japan). Regenerated amorphous cellulose (RAC) was kindly provided by Dr. Peter Westh (DTU, Denmark). Mannose-β-1,4-glucose was purchased from Sweetech (Toulouse, France). 4-Nitrophenyl acetate was obtained from Merck (Rahway, NJ, USA). After overnight reaction at 37 °C, reactions were stopped by adding an equal volume of DNS reagent (ref. 24, 10 g/ L 3,5-dinitrosalicylic acid, 300 g/ L potassium sodium tartrate tetrahydrate, 16 g/L NaOH) followed by heating at 95 ˚C for 10 minutes. Absorbance at 540 nm was then measured and the reducing sugar content was calculated using a standard curve of glucose.

pH profile -The pH profile of the different enzymatic activities was determined by performing triplicate reactions for each enzyme. Reactions contained 5 g/L of specified substrate, 50 mM UB4 buffer with pH values ranging from 3 to 8, and an enzyme concentration appropriate to ensure the reaction remained within the initial rate phase. Reactions were carried out at 37 °C and were terminated using the DNS method as described previously (24).

Kinetic parameters - Kinetic parameters for the GH26|GH5_4 bifunctional enzyme were determined on glucomannan, carob galactomannan, wheat arabinoxylan, and barley mixed-linkage β-glucan. Reactions were performed with varying substrate concentrations and using enzyme concentrations optimized to remain within the initial rates of the reaction, ensuring less than 5% substrate consumption. Assays were conducted at the enzyme's optimal pH for each substrate and at 37 °C. Each reaction was performed in duplicate and monitored in a time course manner. Reactions were stopped by the addition of one volume of bicinchoninic acid (BCA) reagent, and reducing ends were quantified using the BCA method (25), with an internal glucose standard curve. For all conditions, initial rates were calculated for each substrate concentration and plotted against initial substrate concentrations. A Michaelis-Menten model was fitted to the data using OriginPro software, version 2023, (OriginLab Corporation, Northampton, MA, USA).

The activity of the GH26A was assessed on mannobiose and mannotriose. Enzymatic reactions were performed using 0.5 µM enzyme and 5 g/L of substrate in 20 mM UB4 buffer, pH 6, at 37 °C, overnight. Reactions were stopped by heat denaturation at 90 °C for 10 minutes, and the reaction products were analyzed by thin-layer chromatography (TLC).

Carbohydrate esterase -To verify the predicted acetyl esterase activity of CE7, 0.02 µM of the enzyme were assayed for 10 minutes at 37 °C in 200 µL reactions that contained 1 mM 4-nitrophenyl acetate in 50 mM UB4 pH 6 (23). Reactions were monitored spectrophotometrically at the isosbestic point of *p*-nitrophenol at 347 nm. Triplicates were prepared for all enzyme containing samples while two blanks were prepared without enzyme. Initial rates were determined based on product formation and adjusted for the blanks.

Activities of epimerase and phosphorylase - The ability of EpiA to epimerize mannobiose, cellobiose, and mannosyl-β-1,4-glucose was tested by incubating 0.5 µM EpiA with 1 g/L of each substrate in 40 mM UB4 buffer, pH 7, at 37 °C for 1 hr in 200 µL reactions. Reactions were performed in triplicate and stopped by heating to 90 °C for 10 min. Reaction products were analyzed using high-performance anion-exchange chromatography with pulsed amperometric detection (HPAEC-PAD). The activity of GH130 was assayed with mannobiose, cellobiose, and mannosyl-β-1,4-glucose. Reactions were conducted using 0.5 µM GH130 and 5 g/L of substrate in 40 mM UB4 buffer at pH 5 and pH 7 and incubated at 37 °C for 16 hr (23). Reactions were stopped by heat denaturation at 90 °C for 10 minutes and the products were analyzed using HPAEC-PAD.

Analysis of enzymatic reaction products – After termination of the reactions, two volumes of acetonitrile were added. The resulting mixtures were centrifuged for 10 min at 12,000 x g and the supernatants were analyzed by liquid chromatography electrospray ionization mass spectrometry (LC-ESI-MS) on an Amazon SL iontrap (Bruker Daltonics, Bremen, Germany) coupled to an UltiMate 3000 UHPLC from Dionex (Sunnyvale, CA, USA). 5 µL aliquots were injected on a TSKgel amide80 column (2µm, 2mm x 15 cm, Tosoh Bioscience, Tokyo, Japan). Chromatography was performed at 0.3 mL/min at 55 °C on a two-eluent system [eluent A (0.1% formic acid in water), eluent B (acetonitrile)]. The elution profile was as follows: 0–5 min, 75% B; 5–20 minutes, linear gradient to 10% B; 20–23 minutes, isocratic 10% B; 23–30 min, isocratic 75% B. The electrospray was operated in positive ion mode (with UltraScan mode) and a scan range from 100 to 2000 m/z, smart parameter setting of 700 m/z, capillary voltage at 4.5 kV, end plate off-set 0.5 kV, nebulizer pressure at 3.0 bar, dry gas flow at 12 L/min, and dry gas temperature at 280 °C. Products were identified based on their m/z and MS2 fragmentation pattern. Data were analyzed using DataAnalysis 5.3 (Bruker Daltonics,

Bremen Germany). Product quantitation was based on the area of extracted ion chromatograms (Compass TASQ 2.2; Bruker Daltonics, Bremen Germany).

Identification of GH130 reaction products was performed on the same LC-ESI–MS, with the same eluents, but equipped with a porous graphitized carbon column (Hypercarb PGC, 150 mm × 2.1 mm; 3 µm, Thermo Fisher Scientific. Waltham, MA, USA). Chromatography was performed as previously described (26). The MS was operated in alternating mode for simultaneous detection of glucose and mannose-glucose in positive mode and mannose-phosphate in negative mode, with a smart parameter setting of 200 m/z. Quantitation of glucose release was performed using an external standard.

Identification and quantitation of epimerase reaction products were achieved using high-performance anion exchange chromatography with pulsed amperometric detection (HPAEC-PAD) on an ICS6000 system (Dionex Corp., Sunnyvale, California, USA) using a CarboPac PA200 analytical column (4 mm × 250 mm) equipped with a CarboPac PA200 (4 mm × 50 mm) guard column (Dionex Corp., Sunnyvale, California, USA) and a flow rate of 0.2 mL/min at 30 °C. The eluent system comprised Milli-Q water, 1 M sodium hydroxide, and 1 M sodium acetate. The elution profile was as follows: 0-8 min, isocratic 50 mM B, isocratic 0 mM C; 8-12 minutes, isocratic 50 mM B, linear gradient to 300 mM C; 12-20 minutes, isocratic 500 mM B, isocratic 500 mM C; 20-23 minutes, linear gradient to 50 mM B, linear gradient to 0 mM C. Instrument control and quantitation using external standards were performed in Chromeleon 7.1 (Dionex Corp., Sunnyvale, CA, USA).

The esterase activity of CE7 on glucomannan was analyzed by matrix-assisted laser desorption/ionization time-of-flight mass spectrometry (MALDI-TOF MS). Samples were prepared by mixing 2,5-dihydroxybenzoic acid (9 g/L in 70% acetonitrile) and reaction aliquots in a 2:1 ratio on a MTP 384 target plate ground steel BC (Bruker Daltonics GmbH, Bremen, Germany) and dried under a stream of air. Spectra were acquired using a Bruker Autoflex TOF/TOF (Bruker Daltonics GmbH, Bremen, Germany). The instrument was operated in positive acquisition mode and controlled by the FlexControl 3.4 software package. All spectra were obtained using the linear mode using Random Walk and based on the sum of 15,000 shots. The acquisition range used was from m/z 300–5000 Da. Spectra were acquired under the same instrument settings to ensure comparability across replicates. Peak intensities corresponding to selected acetylated and non-acetylated structures (Hex3-Hex6) were extracted using DataAnalysis 5.3 (Bruker Daltonics, Bremen Germany), and relative intensities were calculated by normalizing the signal of each acetylated structure to the intensity of its corresponding non-acetylated counterpart within the same sample. Differences between non-treated samples and CE7-treated samples were assessed using one-way analysis of variance (ANOVA) followed by Tukey's Honest Significant Difference (HSD) test for multiple comparisons. Statistical analyses were performed in R (version 4.3.2) using the 'aov' function for ANOVA and 'TukeyHSD' for post hoc comparisons. Data wrangling and model fitting were performed using the dplyr package (27).

Binding assays of GH26B by affinity-gel electrophoresis - Protein binding was investigated by native polyacrylamide gel electrophoresis in the presence/absence of polysaccharides according to the protocol described in ref. 28. Briefly, 10% polyacrylamide gels were prepared containing glucomannan (0.05% w:v), β-glucan (0.1% w:v), xyloglucan (0.05% w:v), or no polysaccharide. Five and 10 µL of purified GH26B protein at concentrations of 0.5 mg/mL and 1 mg/mL, respectively, were deposited and electrophoresis was conducted at 200 V-

h. Gels were subsequently stained with 0.1% Coomassie Brilliant Blue R-250 in 10% acetic acid, 50% methanol and 40% water.

MOUSE EXPERIMENTS

Husbandry

Mouse experiments were performed using protocols approved by the Washington University Animal Studies Committee. Germ-free C57BL/6J mice were maintained in plastic flexible film isolators (Class Biologically Clean Ltd) at 23 °C with a 12-hour light cycle. The experimental design is described in **Figure 3A**. Two independent mouse experiments were performed and measurements from each experiment were analyzed both separately and in aggregate.

Pregnant C57BL/6J mice originating from trio matings were given *ad libitum* access to an autoclaved breeder chow (Purina Mills; Lab Diet 5021) throughout their pregnancy and until 2 days postpartum. Bacterial strains used in the gavage communities were grown under anaerobic conditions from freezer stocks in WC broth at 37 °C. The strain composition of each gavage mixture is described in **Dataset S7**. The OD_{600} of each strain was used to balance the gavage mixtures to standardize strain representation. All dams received 200 µL of the first bacterial consortium of early-stage colonizers of the Bangladeshi infant gut 4 days postpartum and 200 µL of consortium of later stage colonizers 10 days postpartum. Dams in the *S. copri* colonized arm were gavaged at 7 days postpartum with 200 µL of *S. copri* BgF5_2 grown in WC broth. All gavages were performed using an oral gavage needle (Cadence Science; catalog number 7901).

The free and total monosaccharide composition, plus linkage composition of both animal diets (Mirpur 18, Mirpur-18 + glucomannan) and the glucomannan supplement itself were determined as above and are reported in **Dataset S13**.

Diets were provided to dams and to their weaning and post-weaning pups *ad libitum*. The composition of each diet has been reported previously (2). All diets and ingredients were sterilized by gamma irradiation (30–50 Kgy). Sterility was confirmed by culturing the pellets in Brain-Heart Infusion (BHI) medium supplemented with 0.5% yeast extract (LYBHI) and WC medium under aerobic and anaerobic conditions for 7 days at 37 °C followed by plating on LYBHI and BHI supplemented with 10% sheep blood. Glucomannan was added to the Mirpur-18 diet either by (i) dissolving glucomannan powder (Konjac Root Glucomannan Powder, Best Naturals, Catalog #1771601262T) in water and manually mashing the resulting solution with Mirpur-18 pellets to form a paste prior to feeding or (ii) incorporating the glucomannan powder into the Mirpur-18 diet pellets during their manufacture (Dyets, Inc., Bethlehem, PA).

Statistical analysis of mouse weights

Mouse weights were determined prior to weaning (P18), at weaning (P24 in experiment one, P23 in experiment two), at P35 (after 10-11 days of consuming the Mirpur-18 ± glucomannan diet) and at P53 (after four weeks consuming the Mirpur-18 ± glucomannan diet). Weight data at P53 was divided by weaning weights for each individual mouse to generate a normalized weight. To determine if the combination of *S. copri* colonization and dietary glucomannan consumption resulted significant weight differences between arms over time, the following linear models were employed:

Eq. 1:

$$Weight \sim \beta_{1(sex)} + \beta_{2(P.copri\ colonization)} + \beta_{3(glucomannan\ supplementation)} + \beta_{4(P.copri\ colonization \times glucomannan\ supplementation)} + (1|mouse\ ID)$$

Eq. 2:

$$Weight_{normalized} \sim \beta_{1(weight\ at\ weaning)} + \beta_{2(sex)} + \beta_{3(P.copri\ colonization)} + \beta_{4(glucomannan\ supplementation)} + \beta_{5(P.copri\ colonization \times glucomannan\ supplementation)}$$

Models were fitted and significance determined using the lmerTest package in R. The significance of the '*S. copri* Colonization x Glucomannan supplementation term' was used to determine if there were statistical interactions between *S. copri* colonization and glucomannan diet supplementation. Data were analyzed separately within each experimental replicate separately, then by including data from both experimental replicates. In addition to the time course analyses, we also analyzed the P35 and P53 data in cross-sectional analysis using linear models similar to the above.

Biospecimen collection

Fecal samples collected along the course of the experiment, plus biospecimens collected from mice when they were euthanized (without prior fasting), were snap frozen in liquid nitrogen and stored at −80 °C before use. Prior to flash freezing, small intestines were divided into equal thirds using a razor blade. The proximal third of the small intestine was arbitrarily defined as the duodenum, the middle third as the jejunum, and the distal third as the ileum. The distal 5 cm of the jejunum and ileum were further divided into 1 cm segments. The proximal 3 cm of the mouse colon was divided into 1 cm segments as well. The second most distal 1 cm jejunal and ileal segments and the second most proximal 1 cm jejunal and ileal segments were used for mass spectrometric analysis. Cecal contents were collected and aliquoted into ~80 mg portions using 10 μL inoculating loops (VWR; catalog number: 76544-926). The tip of each loop was removed using wire cutters, deposited into a collection tube and flash frozen in liquid nitrogen.

Measurement of the absolute abundances of bacterial strains in the mouse gut

The absolute abundances of bacterial strains were determined using previously described methods with minor modifications (2). In brief, a 'spike-in' mixture containing *Alicyclobacillus acidiphilus* DSM 14558 (2.22×10^8 cells/mL; 15 μL per sample) and *Rhizobium radiobacter* DSM 30147 (9.93×10^8 cells/mL; 30 μL per sample) was added to weighed frozen fecal or cecal samples prior to isolation of total nucleic acid isolation and preparation of barcoded libraries for shotgun sequencing. Sequencing was performed on a total of 287 samples across two runs (n = 128 and 159 samples per run, respectively) using an Illumina NextSeq instrument. Samples were sequenced to an average depth of $1.04 \times 10^6 \pm 0.13 \times 10^6$ reads in the first run and $1.78 \times 10^6 \pm 1.04 \times 10^6$ reads in the second. Bacterial abundances were determined using community profiling by sequencing (COPRO-Seq, ref. 29). The resulting count table was imported into R (v4.1.2). We calculated the absolute abundance of a given $strain_i$ in a given $sample_j$ in reference to the abundances of the spike-in organisms *A. acidiphilus* (Aa) and *R. radiobacter* (Rr) genomes using the following equation:

Eq. 3:

$$Abundance\ of\ strain_{i,j} = 0.5 \times \left(\frac{counts_{i,j} \times Aa\ cells\ added_j}{Aa\ counts_j \times sample\ weight_j} + \frac{counts_{i,j} \times Rr\ cells\ added_j}{Rr\ counts_j \times sample\ weight_j} \right)$$

The output of this equation is genome equivalents per gram of feces. For statistical testing, this metric was $\log_{10}$ transformed. The significance of differences in *S. copri* or other organism abundances at P25, P35, and P53 was determined using the Mann-Whitney U test or between timepoints within a given group of animals using Paired Mann-Whitney tests. *P*-values were adjusted to control for false discovery rate using the Benjamini-Hochberg method. Statistical significance was defined as adjusted *P*-values < 0.05.

Transcriptional profiling of *S. copri* and other bacterial strains from *in vivo* biospecimens

RNA was isolated from fecal and cecal contents collected at the end of the experiment. Complementary DNA (cDNA) libraries were generated from isolated RNA samples using the 'Total RNA Prep with Ribo-Zero Plus' kit (Illumina). Barcoded libraries were sequenced (Illumina NovaSeq instrument). A total of 77 cecal samples were sequenced to a depth of $2.00 \times 10^7 \pm 7.6 \times 10^6$ paired-end 150 nt reads (first run: $1.28 \times 10^7 \pm 1.3 \times 10^6$ reads; second run $2.67 \times 10^7 \pm 3.9 \times 10^6$ reads). 76 fecal samples were sequenced to a depth of $2.37 \times 10^7 \pm 1.11 \times 10^7$ paired-end 150 nt reads (first run: $1.48 \times 10^7 \pm 3.9 \times 10^6$ reads; second run $3.21 \times 10^7 \pm 8.9 \times 10^6$ reads).

Both the metatranscriptomic (MTX, cDNA) library reads and the metagenomic (MGX, DNA) library reads were mapped to the genomes of all colonized bacterial strains. To do so, reads were trimmed to remove low quality bases and adapter sequences using *trimgalore* (v0.6.4; ref. 15) and further filtering out trimmed reads less than 100 bp. Reads aligning to the mouse genome (GRCm39) were identified and removed using *bowtie2* (v2.4.2; ref. 30). Separately, both MTX and MGX filtered reads were pseudoaligned to an index containing all open reading frames from the genomes of all gavaged bacteria using *kallisto* (v0.48.0; ref. 16). This effort yielded gene-by-samples count tables for both MGX and MTX libraries. Counts mapping to ribosomal RNA loci predicted by prokka (31) in any of the genomes were filtered out to prevent skewing of library size normalization. Samples from both experiments were divided into fecal and cecal sample sets and then further divided into two sets of comparisons: (i) glucomannan-supplemented versus glucomannan-unsupplemented in animals colonized with *S. copri* and (ii) glucomannan-supplemented versus glucomannan-unsupplemented in animals not colonized with *S. copri.*

Differential expression testing was performed using *MTXmodel* (32). *MTXmodel* is a generalized-linear model-based approach for differential expression testing of microbial communities that controls false positives due to changes in underlying organism abundances. Both DNA and cDNA library counts tables for each of the comparisons described above were used as input. Each comparison was run using 'diet' as the single fixed effect in the model design. Transcripts were deemed significantly differentially expressed (i.e., had 'diet' coefficients that were significantly different from 0) if their Benjamini-Hochberg adjusted *P*-value was less than 0.1.

SUPPLEMENTARY FIGURES:

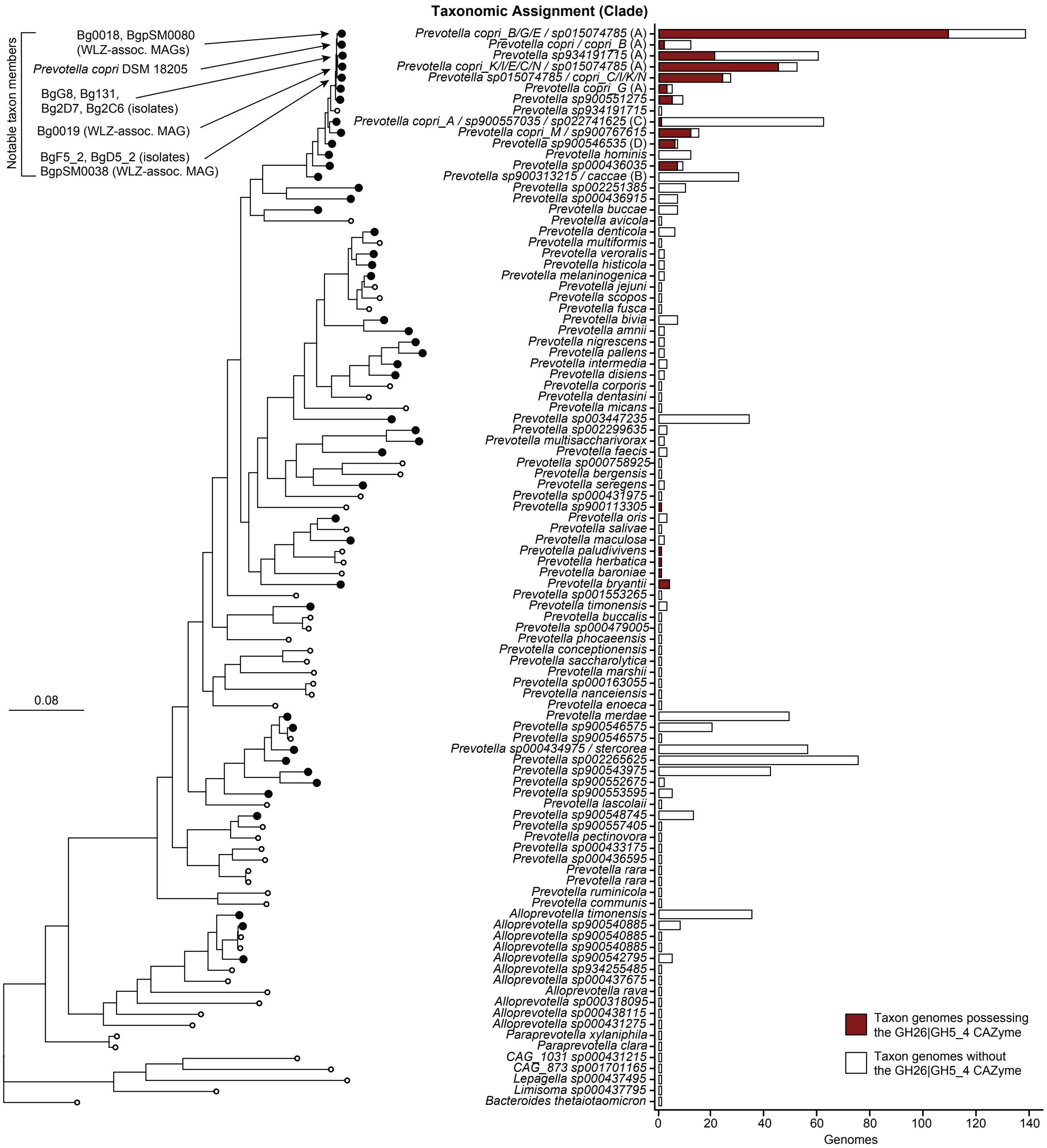

Fig. S1. Phylogenetic distribution of the GH26|GH5_4 CAZyme among *Prevotella*/*Segatella* MAGs and isolate genomes. Marker gene sequences were extracted from each genome, aligned and used to generate a phylogenic tree (left). Tree nodes were collapsed (black circles) based on tree topology and GTDB-based genome taxonomic assignment. In addition, the Bg0019 GH26|GH5_4 reference sequence was searched against the same genomes using tBLASTn. The results were filtered to alignments encompassing ≥700 of the 786 amino acids in the MAG

Bg0019 query sequence and used to annotate the tree. Alignments displaying ≥ 50% amino acid similarity satisfied our criteria for possessing the bimodular CAZyme, and the prevalence of the gene encoding the enzyme was determined within each collapsed node (right). The scale bar at left indicates phylogenetic distance.

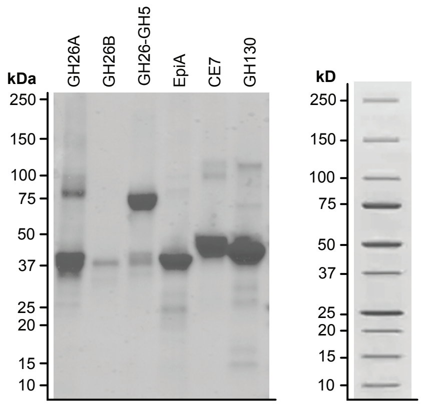

Fig. S2. SDS-PAGE of the enzymes associated with PUL 7 after expression in *E. coli* and subsequent purification.

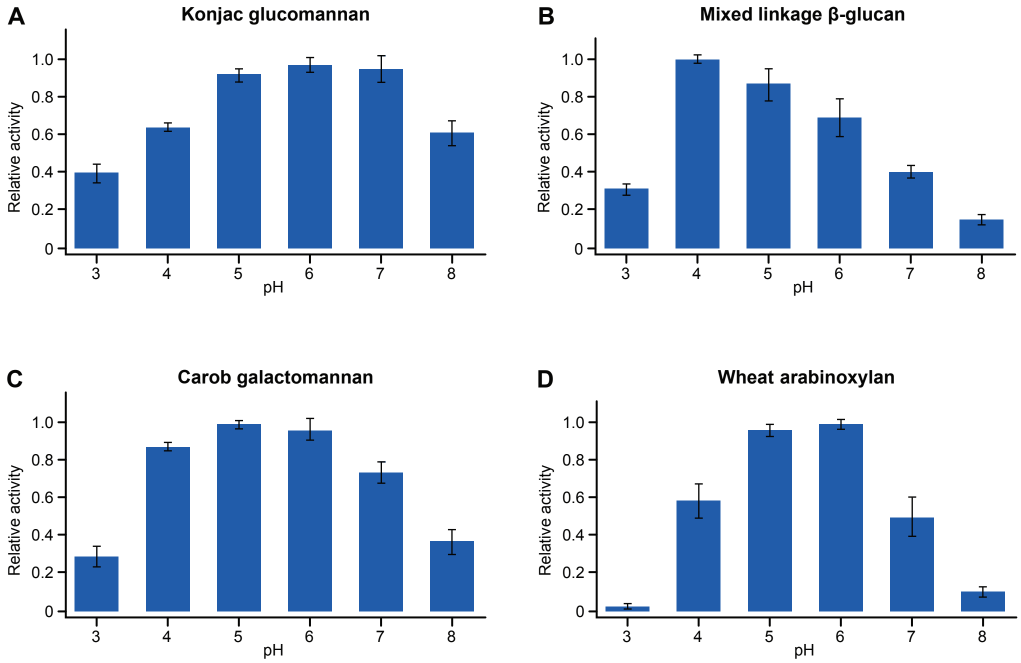

E

Substrate	Km (g/L)	kcat (1/s)	kcat/Km (L/g x s)
Konjac glucomannan	52.8 ± 3.8	55.6	0.95 ± 0.17
Mixed linkage β-glucan	68.5 ± 3.2	152.2	0.45 ± 0.06
Carob galactomannan	66.0 ± 8.5	19.1	3.45 ± 0.94
Wheat arabinoxylan	1.06 ± 0.04	0.7	1.46 ± 0.15

Fig. S3. Reaction characteristics of the GH26|GH5_4 enzyme acting on various substrates. (**A-D**) Relative activities of the GH26|GH5_4 enzyme at pH 3-8 for the substrates glucomannan, (panel A), mixed linkage β-glucan, (panel B) galactomannan (panel C) and arabinoxylan (panel D). Activity was determined by assaying reducing end concentrations using the BCA method (25). (**E**) Kinetics of degradation of glucomannan, β-glucan, galactomannan and arabinoxylan by the GH26|GH5_4 enzyme.

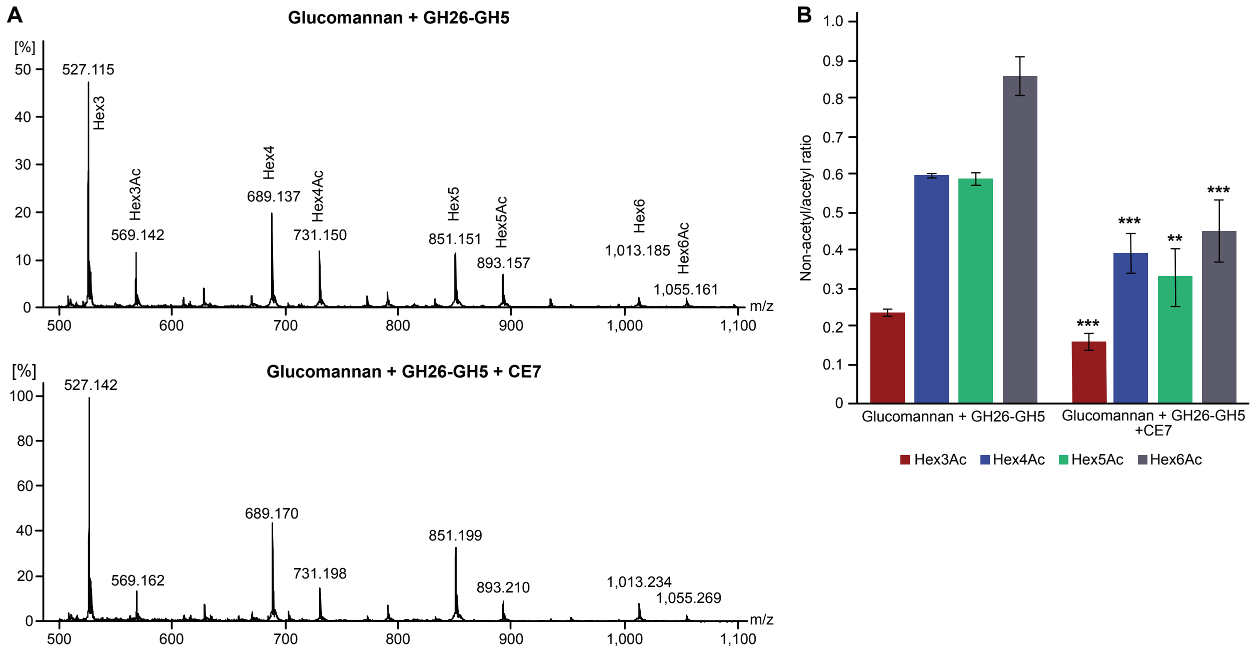

Fig. S4. Glucomannan degradation products. (**A**) LC-ESI-MS product profiles of glucomannan incubated with (upper) the GH26|GH5_4 bimodular enzyme from PUL 7 or (lower) the GH26|GH5_4 and CE7 enzymes. (**B**) The ratio between non-acetylated and acetylated structures in reactions containing GH26|GH5_4 treated glucomannan with or without CE7, based on signal intensity. Differences between non-treated samples and CE7-treated samples were assessed using ANOVA followed by Tukey's HSD. *, $P < 0.05$; **, $P < 0.01$; ***, $P < 0.005$.

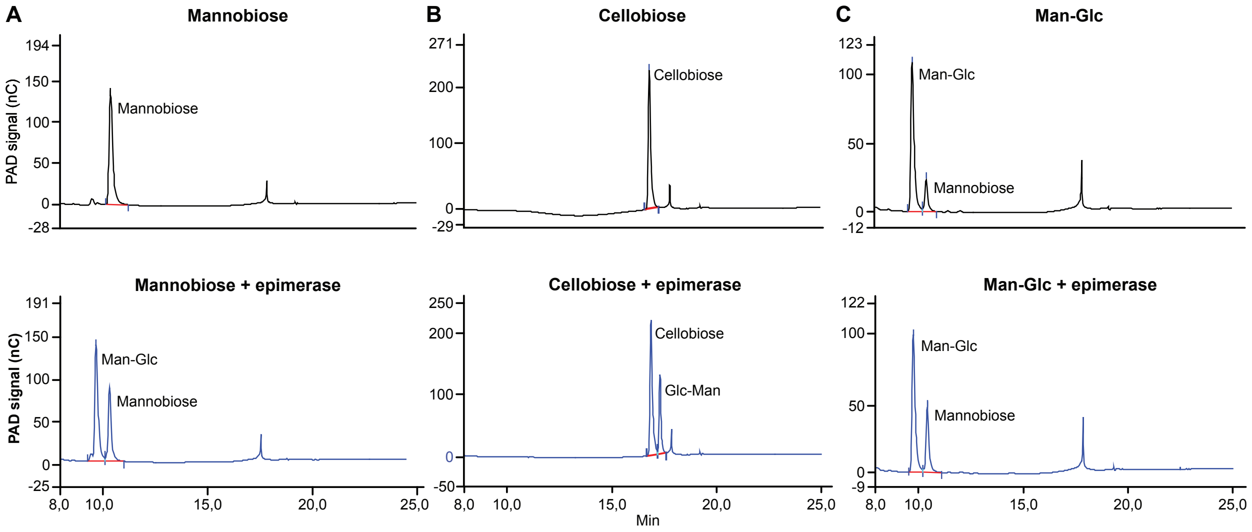

Fig. S5. HPAEC-PAD chromatograms showing the epimerization activity of EpiA on different substrates. (**A-C**) Epimerization was determined for substrates including (**A**) mannobiose, (**B**) cellobiose and (**C**) mannosyl-β-1,4-glucose. Top chromatograms display the substrates alone, while the bottom chromatograms describe analysis of reaction products after incubation with EpiA.

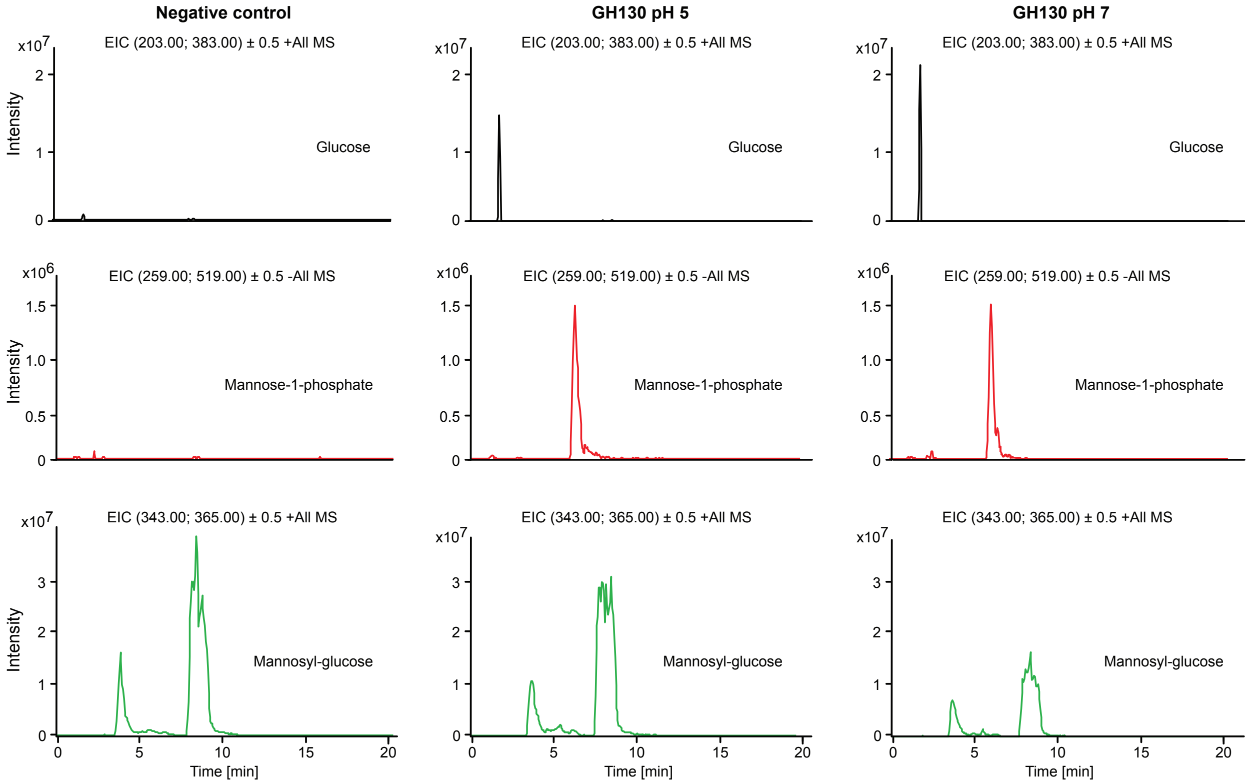

Fig. S6. LC-ESI-MS product chromatograms of the GH130 CAZyme. Tested substrates correspond to glucose ($[M+Na]^+$, $[2M+Na]^+$; upper), mannose-1-phosphate ($[M-H]^-$, $[2M-H]^-$; middle), and mannosyl-glucose ($[M+H]^+$, $[M+Na]^+$; lower). Treatments include no enzyme (left), GH130 at pH 5 (middle), and GH130 at pH 7 (right).

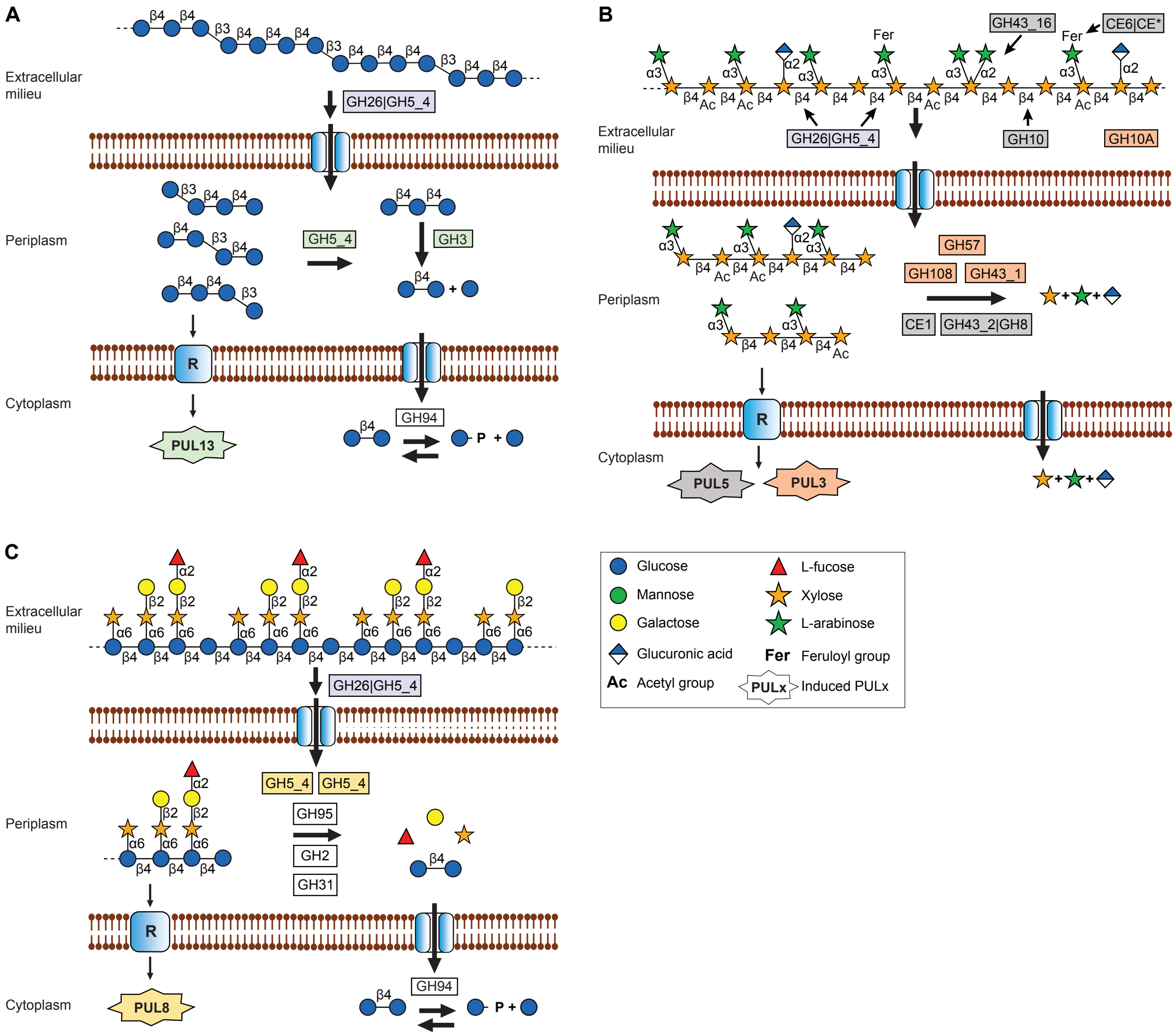

Fig. S7. Proposed mechanisms of degradation of MDCF-2-related polysaccharides by *S. copri* BgF5_2 PULs. (A-C). Proposed schematics of complete β-glucan degradation (panel A), arabinoxylan degradation (panel B) and xyloglucan degradation (panel C) in the cultured BgF5_2 isolate based on the biochemically-confirmed activities of its PUL 7 and PUL 8 CAZymes, their predicted cellular localization as determined by the presence or absence of signal peptides, and the predicted activities of other PUL and non-PUL associated enzymes. PUL naming conventions are those of MAG Bg0019 (see **Table 1** for details).

DATASET LEGENDS

Dataset S1: Carbohydrate-active enzymes encoded by select polysaccharide utilization loci in the genome of MAG Bg0019. (**A**) Proteins encoded by PUL 7 and PUL 8 of MAG Bg0019. (**B**) Conservation of PUL-encoded genes between MAG Bg0019 PUL 7 and PUL 8 and additional *Segatella copri* isolate genomes. (**C**) *In vitro* growth data for strains BgF5_2 and DSM 18205 in carbohydrate-deficient defined medium supplemented with glucomannan or glucose.

Dataset S2: Bacteroidota genomes evaluated for the phylogenetic distribution of the multifunctional GH26|GH5_4 CAZyme.

Dataset S3: Differential gene expression for *S. copri* BgF5_2 grown in PCDM-glucomannan versus PCDM-glucose. (**A**) Gene-level differential expression results (DESeq2). (**B**) Gene Set Enrichment Analysis of PUL differential gene expression *in vitro* between *S. copri* grown in glucomannan compared to glucose. (**C**) Gene Set Enrichment Analysis of metabolic pathway differential gene expression *in vitro* between *S. copri* grown in glucomannan compared to glucose.

Dataset S4: Monosaccharide and linkage quantification from PCDM-glucomannan incubated with or without *S. copri* BgF5_2. (**A**) Total monosaccharide (mg/mL of supernatant). (**B**) Free monosaccharide (mg/mL of supernatant). (**C**) Linkages (Arbitrary units).

Dataset S5: Biochemical activity of expressed and purified PUL 7 CAZymes *in vitro*. (**A**) Correspondence between CAZyme domains present in PUL 7 enzymes, predicted target linkages and substrates. (**B**) Increase in reducing end concentration in reactions containing the indicated glycan substrate and individual purified PUL 7 CAZymes.

Dataset S6: Concentrations of oligomeric products generated by *in vitro* digestion of polysaccharides by purified PUL 7 CAZymes. (**A**) Oligomer product profiles of substrate digestion by GH26|GH5_4 and GH26A CAZymes. (**B**) Oligomer product profiles of xylan and xyloglucan digestion by GH26|GH5_4 and GH5_4 CAZymes. (**C**) Oligomer product profiles of glucomannan and mannan digestion by GH26A as determined by HPAEC-PAD.

Dataset S7: Bacterial strains used in the defined community gnotobiotic mouse experiments.

Dataset S8: Gnotobiotic mouse body weights at four weeks after weaning.

Dataset S9: Gnotobiotic mouse fecal and cecal microbiota profiling. (**A**) Fecal *S. copri* BgF5_2 abundances as determined by COPRO-Seq analysis. (**B**) Fecal microbial community member abundances as determined by COPRO-Seq analysis of animals whose communities included *S. copri*. (**C**) Cecal microbial community abundances as determined by COPRO-Seq analysis of animals whose microbial communities included *S. copri*. (**D**) Fecal microbial community abundances as determined by COPRO-Seq analysis of animals whose microbial communities did not include *S. copri*. (**E**) Cecal microbial community abundances as determined by COPRO-Seq analysis of animals whose microbial communities did not include *S. copri*.

Dataset S10: Differential gene expression for *S. copri* BgF5_2 across two gnotobiotic animal studies. (**A**) Differential expression results for cecal samples. (**B**) Differential expression results for fecal samples. (**C**) Gene Set Enrichment Analysis of differential gene expression of *S. copri* in the cecal contents or feces of mice fed Mirpur-18 + Glucomannan versus Mirpur-18 alone

Dataset S11: Differential gene expression of entire microbial community *in vivo*. (**A**) Summary of differential expression analysis of non-*S. copri* community members in cecal and fecal samples. (**B**) Differential expression analysis of non-*S. copri* community members in cecal samples. (**C**) Differential expression analysis of non-*S. copri* community members in fecal samples.

Dataset S12: Cecal monosaccharide and linkage measurements. (**A**) Total monosaccharides. (**B**) Free monosaccharides. (**C**) Linkages.

Dataset S13: Monosaccharide and linkage measurements in animal diets. (**A**) Total monosaccharides. (**B**) Free monosaccharides. (**C**) Linkages.

SI REFERENCES

1. M. C. Hibberd, *et al.*, Bioactive glycans in a microbiome-directed food for children with malnutrition. *Nature* **625**, 157–165 (2024).

2. H.-W. Chang, *et al.*, *Prevotella copri* and microbiota members mediate the beneficial effects of a therapeutic food for malnutrition. *Nat. Microbiol.* **9**, 922–937 (2024).

3. T. Paysan-Lafosse, *et al.*, InterPro in 2022. *Nucleic Acids Res.* **51**, D418–D427 (2022).

4. D. A. Rodionov, *et al.*, Micronutrient Requirements and Sharing Capabilities of the Human Gut Microbiome. *Front Microbiol* **10**, 1316 (2019).

5. R. D. Olson, *et al.*, Introducing the Bacterial and Viral Bioinformatics Resource Center (BV-BRC): a resource combining PATRIC, IRD and ViPR. *Nucleic Acids Res.* **51**, D678–D689 (2022).

6. E. W. Sayers, *et al.*, Database resources of the National Center for Biotechnology Information in 2025. *Nucleic Acids Res.* **53**, D20–D29 (2024).

7. D. H. Parks, M. Imelfort, C. T. Skennerton, P. Hugenholtz, G. W. Tyson, CheckM: assessing the quality of microbial genomes recovered from isolates, single cells, and metagenomes. *Genome Res* **25**, 1043–1055 (2015).

8. M. N. Price, P. S. Dehal, A. P. Arkin, FastTree 2 – Approximately Maximum-Likelihood Trees for Large Alignments. *PLoS ONE* **5**, e9490 (2010).

9. E. Paradis, K. Schliep, ape 5.0: an environment for modern phylogenetics and evolutionary analyses in R. *Bioinformatics* **35**, 526–528 (2018).

10. P.-A. Chaumeil, A. J. Mussig, P. Hugenholtz, D. H. Parks, GTDB-Tk: a toolkit to classify genomes with the Genome Taxonomy Database. *Bioinformatics* **36** (6) 1925-1927 (2019).

11. G. Yu, Using ggtree to Visualize Data on Tree-Like Structures. *Curr. Protoc. Bioinform.* **69**, e96 (2020).

12. E. Drula, *et al.*, The carbohydrate-active enzyme database: functions and literature. *Nucleic Acids Res* **50**, D471-D577 (2021).

13. S. F. Altschul, W. Gish, W. Miller, E. W. Myers, D. J. Lipman, Basic local alignment search tool. *J Mol Biol* **215**, 403–410 (1990).

14. J. Li, *et al.*, A versatile genetic toolbox for *Prevotella copri* enables studying polysaccharide utilization systems. *EMBO J.* **40**, e108287 (2021).

15. F. Krueger, F. James, P. Ewels, E. Afyounian, B. Schuster-Boeckler, TrimGalore. Available at: https://zenodo.org/record/5127899#.YQxVhFNKiDU. Accessed 1/10/2023.

16. N. L. Bray, H. Pimentel, P. Melsted, L. Pachter, Near-optimal probabilistic RNA-seq quantification. *Nat Biotechnol* **34**, 525–527 (2016).

17. M. I. Love, W. Huber, S. Anders, Moderated estimation of fold change and dispersion for RNA-seq data with DESeq2. *Genome Biol* **15**, 550 (2014).

18. G. Korotkevich, *et al.*, Fast gene set enrichment analysis. *BioRxiv* 060012 (2021). https://doi.org/10.1101/060012. Accessed 25/10/2023.

19. G. Xu, M. J. Amicucci, Z. Cheng, A. G. Galermo, C. B. Lebrilla, Revisiting monosaccharide analysis – quantitation of a comprehensive set of monosaccharides using dynamic multiple reaction monitoring. *Analyst* **143**, 200–207 (2017).

20. M. J. Amicucci, *et al.*, A rapid-throughput adaptable method for determining the monosaccharide composition of polysaccharides. *Int. J. Mass Spectrom.* **438**, 22–28 (2019).

21. A. G. Galermo, *et al.*, Liquid Chromatography–Tandem Mass Spectrometry Approach for Determining Glycosidic Linkages. *Anal Chem.* **90**, 13073–13080 (2018).

22. A. G. Galermo, E. Nandita, J. J. Castillo, M. J. Amicucci, C. B. Lebrilla, Development of an Extensive Linkage Library for Characterization of Carbohydrates. *Anal. Chem.* **91**, 13022–13031 (2019).

23. D. Brooke, N. Movahed, B. Bothner, Universal buffers for use in biochemistry and biophysical experiments. *AIMS Biophys.* **2**, 336–342 (2015).

24. G. L. Miller, Use of Dinitrosalicylic Acid Reagent for Determination of Reducing Sugar. *Anal. Chem.* **31**, 426–428 (1959).

25. G. Arnal, M. A. Attia, J. Asohan, H. Brumer, "A Low-Volume, Parallel Copper-Bicinchoninic Acid (BCA) Assay for Glycoside Hydrolases" in *Methods Mol Biol* (Springer New York, 2017), pp. 3–14.

26. B. Zeuner, *et al.*, Substrate specificity and transfucosylation activity of GH29 α-l-fucosidases for enzymatic production of human milk oligosaccharides. *Nat Biotechnol.* **41**, 34–45 (2018).

27. H. Wickham *et al.*, dplyr: A Grammar of Data Manipulation. https://dplyr.tidyverse.org. (2025).

28. D. Cockburn, C. Wilkens, B. Svensson, Affinity Electrophoresis for Analysis of Catalytic Module-Carbohydrate Interactions. *Methods Mol. Biol.* **1588**, 119–127 (2017).

29. M. C. Hibberd, *et al.*, The effects of micronutrient deficiencies on bacterial species from the human gut microbiota. *Sci Transl Med* **9**, eaal4069 (2017).

30. B. Langmead, S. L. Salzberg, Fast gapped-read alignment with Bowtie 2. *Nat Methods* **9**, 357–359 (2012).

31. T. Seemann, Prokka: rapid prokaryotic genome annotation. *Bioinformatics* **30**, 2068-2069 (2014).

32. Y. Zhang, K. N. Thompson, C. Huttenhower, E. A. Franzosa, Statistical approaches for differential expression analysis in metatranscriptomics. *Bioinformatics* **37**, i34–i41 (2021).
